# Addressing consistency and demand uncertainty in the Home Care planning problem

**DOI:** 10.1007/s10696-021-09412-z

**Published:** 2021-04-04

**Authors:** Paola Cappanera, Maria Grazia Scutellà

**Affiliations:** 1 Dipartimento di Ingegneria dell’Informazione(DINFO), University of Florence, Florence, Italy; 2 Dipartimento di Informatica, University of Pisa, Pisa, Italy

## Abstract

Optimizing Home Care Services is receiving a great attention in Operations Research. We address arrival time consistency, person-oriented consistency and demand uncertainty in Home Care, while jointly optimizing assignment, scheduling and routing decisions over a multiple-day time horizon. Consistent time schedules are very much appreciated by patients who, in this setting, are very sensitive to changes in their daily routines. Also person-oriented consistency positively impacts on service quality, guaranteeing that almost the same set of caregivers take care of a patient in the planning horizon. Demand uncertainty plays a pivotal role, too, since both the set of patients under treatment and their care plan can change over time. To the best of our knowledge, this is the first paper dealing with all these aspects in Home Care via a robust approach. We present a mathematical model to the problem, and a pattern-based algorithmic framework to solve it. The framework is derived from the model via decomposition, i.e. suitably fixing the scheduling decisions through the concept of pattern. We propose alternative policies to generate patterns, taking into account consistency and demand uncertainty; when embedding them in the general framework, alternative pattern based algorithms originate. The results of a rich computational experience show that introducing consistency and demand uncertainty in pattern generation policies is crucial to efficiently compute very good quality solutions, in terms of robustness and balancing of the caregiver workload. In addition, a comparison with a simpler model, where no kind of consistency is imposed, shows the importance of considering consistency in pursuing a valuable patient-centered perspective, with a positive effect also on the efficiency of the solution approach.

## Introduction

The design of efficient Home Care Services is an area of study which is receiving increasing attention in Operations Research, motivated by the ever increasing age of population and the consequent need to reduce hospitalization costs. While cost containment is an objective usually pursued in many private as well as public companies, in the Home Care setting the design of patient-centered services definitely deserves investigation and as a result, may also entail an increase of profitability. The term *Home Care Services* refers to medical, paramedical and social services that may be delivered to patient homes. The provision of Home Care services has never been as relevant as in this period: indeed, it is crucial both in the COVID-19 era to cover overwhelming requirements and in the post COVID-19 phase to support all those activities that during the acute phase of the pandemic have been reduced to the minimum such as non-urgent surgery, outpatient clinics and treatments, and diagnostic tests. Worldwide, healthcare systems are facing an unprecedented scenario and new challenges are being posed (Bryant et al. (2020), Coloma and Nicols (2020)).

Home Care Services need to address many complex decisions such as caregiver-to-patient assignment, scheduling of patient requests, and caregiver routing. Such decisional aspects have usually been considered separately in the literature, essentially by means of heuristic approaches (e.g. Eveborn et al. (2006), Liu et al. (2014), Nickel et al. (2012), Redjem and Marcon (2016)). On the other hand, the number of exact approaches which deal simultaneously with the different planning aspects in Home Care is still rather small. We mention Gamst and Jensen (2012), Rasmussen et al. (2012) (on a daily perspective), Trautsamwieser and Hirsch (2014), and Cappanera and Scutellà (2015) where the concept of *pattern*, i.e. a template for scheduling multiple visits, has been introduced to coordinate several decisions in a multiple-day planning horizon. Patterns proved useful also in designing efficient decomposition algorithms Yalçındağ et al. (2016). We refer to Di Mascolo et al. (2017), Cissé et al. (2017) and Fikar and Hirsch (2017) for recent and comprehensive reviews on both heuristic and exact approaches to planning decisions in Home Care.

Although the literature on Home Care Services is now wide, data uncertainty has been taken into account only recently. In this context, addressing *demand uncertainty* (Cappanera et al. (2018)) plays a pivotal role since both the set of patients under treatment and their care plan can change over time due to unexpected events. Thus, patient or visit cancellations as well as the insertion of new patients are likely to occur. Clearly, such uncertain events could be managed via a deterministic approach, by either disregarding the uncertain requests and optimizing only on the certain ones, or considering all the patient requests, including the uncertain ones, as certain. The first approach, that ignores the uncertain requests, would certainly produce cheaper solutions. But if any of these uncertain requests would realize later on during the time horizon, likely these might not be served, giving rise to service disruptions. The second approach, instead, would produce very costly solutions that might turn out to be strongly underused. Furthermore, trying to serve all the uncertain requests could lead to unfeasibility. This is why managing demand uncertainty via Robust Optimization, which offers a good trade-off between solution cost and care plan quality, appears to be particularly relevant in the Home Care context. In addition, a very few works have addressed *consistency* in Home Care. Consistency means service regularity and it is crucial also in related problems which are intimately connected to Home Care, like Field Service and more generally Vehicle Routing Problems (VRP). In particular, in VRP three main kinds of consistency are addressed (Kovacs et al. (2014)): (*i*) arrival time consistency - visit each patient at about the same time, (*ii*) person-oriented consistency - use the same subset of operators for all the visits of a patient and (*iii*) delivery consistency - deliver always the same quantity or with the same frequency.

Arrival time consistency and person-oriented consistency have nowadays a preeminent role also in Home Care. Time consistent schedules are very much appreciated by patients who, in this setting, are often fragile and thus particularly sensitive to changes in their daily routines. When arrival time consistency is imposed, all the visits in the care plan of a patient are done at about the same time during the planning horizon. This kind of service regularity offers a series of valuable advantages to patients, to their relatives and to the service provider. In fact, arrival time consistency: (*i*) positively impacts the trust the patient places in the service provider, (*ii*) allows people around the patient (relatives and caregivers) to better organize and plan their own activities, and (*iii*) makes it possible to take advantage of flexibility in designing the service. Indeed, a different and less flexible way to guarantee time consistency might be represented by time windows imposed on the visit schedule: the patients themselves might express a preference (soft constraint) or a hard constraint on the time slot in which visits should occur. However, when imposing time window constraints, the service provider may fail to get a feasible solution that accommodates all the patients’ requests and, consequently, a negotiation process with patients must start to relax their requests possibly incurring in penalties for the provider and, ultimately, raising patient dissatisfaction. In contrast, when arrival time consistency is assured, the patients take for granted that all their visits will occur at about the same time, even if they do not know at what time, and the service provider is thus able to exploit the flexibility deriving from choosing the time slot in which visits will be given. This is especially reasonable for categories of patients like the palliative ones, considered in our computational study. Also notice that, although patients do not express their preferences on time of visits, the optimized schedule clearly contains information on time, thus allowing the service provider to advice patients well in advance.

Also person-oriented consistency positively impacts on service quality, both from the patient and the caregiver side. Indeed, person-oriented consistency, often referred to as *care continuity* in the Home Care literature (Borsani et al. (2006), Duque et al. (2015)), guarantees that almost the same set of caregivers take care of a patient in the planning horizon. The communication between patients and caregivers is therefore simplified: caregivers who visit patients on a regular basis acquire information about their preferences, thus making it possible to better organize working activities and to provide a high quality service. In addition, because of the physical intimacy, patients feel comfortable when they receive service by known caregivers.

The aim of this paper is to consider both consistency and demand uncertainty in Home Care, by extending the Robust Home Care Problem introduced in Cappanera et al. (2018). Precisely, the main contributions can be summarized as follows: (1) we define a new problem in Home Care, i.e. the Consistent Robust Home Care problem, where arrival time consistency, person-oriented consistency and demand uncertainty are addressed while jointly optimizing assignment, scheduling and routing over a multiple-day time horizon; (2) we present a mathematical model and a matheuristic to the Consistent Robust Home Care problem, suitably fixing scheduling decisions within the model through the concept of pattern; (3) we propose alternative pattern generation policies, explicitly taking into account consistency issues in addition to demand uncertainty; when embedding them in the general framework, alternative pattern based matheuristics originate; (4) we present the results of a wide computational experience, on newly generated instances based on real data, showing that the pattern based approach seems to be a viable tool to successfully address the Consistent Robust Home Care problem, especially when the pattern generation policies encompass consistency and demand uncertainty; in fact, some of the algorithms are able to efficiently compute very good quality solutions, particularly in terms of robustness; (5) we compare the proposed algorithms with a simpler approach, where no consistency is imposed, showing the value of considering consistency from a patient-centered perspective; in fact, not considering consistency has a negative impact on the solution quality, in terms of number of patients who are visited by more than one caregiver, and also in terms of number of patients with visits scheduled in different time slots during the planning horizon.

To the best of our knowledge, the novelty of both the model and the algorithm proposed still remain also when the related context of Consistent VRP is considered where as far as we know, arrival time consistency, person-oriented consistency and demand uncertainty issues have never been addressed together via robust optimization.

The plan of the paper is the following. After a review of the literature addressing uncertainty or consistency in Home Care and in some related problems, provided in Sect. 2, the Consistent Robust Home Care Problem is introduced in Sect. 3. Sect. 4 presents the robust mathematical model we propose to formulate the problem. Section 5 outlines the pattern based algorithmic framework used to solve the problem, which relies on the mathematical model in Sect. 4. In particular, the Sects. 5.1.1 and 5.1.2 present alternative consistent pattern generation policies which allow to obtain alternative consistent algorithms from the general framework outlined in Sect. 5. Section 6 presents the results of a rich computational experience aimed at evaluating the proposed algorithms in terms of efficiency and quality of the determined solutions. Section 7 reports computational results showing the value of considering consistency on the patient perspective. Section 8 concludes the paper.

## Literature review: uncertainty and consistency in Home Care

Here we shall describe the main results from the literature concerning data uncertainty in Home Care (Sect. 2.1) and consistency in Home Care (Sect. 2.2). Papers dealing with uncertainty together with some forms of (relaxed) consistency will be presented in Sect. 2.3, with the exception of those where the only form of consistency addressed is the care continuity one, which will be mentioned in 2.1. The review will include also papers dealing with consistency in the strictly related context of VRP; this will be provided in Sect. 2.4.

### Uncertainty in Home Care

As outlined before, the literature addressing data uncertainty in Home Care is quite recent, and therefore there are a few works on this subject. In Carello and Lanzarone (2014) the problem of assigning a set of patients to a set of caregivers over a time horizon is considered imposing care continuity, i.e. exactly one caregiver must be assigned to each patient, and assuming that the amount of working time required by each patient is uncertain. A cardinality-constrained robust assignment model is proposed and tested on real-life instances. Also, a robust assignment model which includes the time-dependency of the patient demands, and which is based on the implementor-adversarial framework, is presented in Carello et al. (2017). An implementor/adversary algorithm is used also in Holte and Mannino (2013) for a cyclic robust scheduling problem arising in health care. Moreover, a real-time scheduling model is proposed in Du et al. (2019) to minimize the total required scheduling time in case of unexpected events, together with an improved memetic algorithm to optimize the model. Notice that only assignment or scheduling decisions are addressed in the above mentioned papers. Scheduling and routing in Home Care in the case of stochastic service times are addressed in Yuan et al. (2014). A stochastic programming model with recourse is proposed, which is then transformed into a set partitioning problem and an elementary shortest path problem with resource constraints. A branch and price approach is designed and validated by means of numerical experiments. The case of stochastic service times is also addressed in Rodriguez et al. (2015), where the authors propose a two-stage approach, based on integer linear stochastic programming, to deal with staff dimensioning in Home Care, and in Liu et al. (2018), where a branch and price algorithm is proposed by considering stochastic travel times in addition to stochastic service times. Uncertainty on the availability of the caregivers is considered in Nguyen et al. (2015), where a matheuristic is proposed to handle the case where caregivers are unexpectedly unable to operate. More recently, demand uncertainty in Home Care is addressed in Hewitt et al. (2016) by estimating the demands, at the beginning of the time horizon, starting from some historical data. The strategy of optimizing for the entire time horizon, using such demand estimates and imposing care continuity, is compared to a rolling horizon strategy, where demands are updated dynamically as soon as they reveal, using an iterative heuristic approach. Scheduling decisions are not addressed. Demand uncertainty and care continuity are managed also in Cappanera et al. (2018) where, in addition, patient assignment, scheduling of patient requests and caregiver routing are jointly optimized via a special case of $\Gamma$-robustness (Bertsimas and Sim (2004)).

Finally, considering a related application context, a robust optimization model for dispatching technicians under stochastic service times is proposed in Souyris et al. (2013) within a branch and price framework. Moreover, a multi-period technician scheduling problem with experience-based service times and stochastic customers is addressed in Chen et al. (2012).

### Consistency in Home Care

By considering the Home Care studies, only a few papers have addressed a kind of consistency different from the care continuity one, which on the contrary has been widely taken into account. The focus here is on this restricted set of papers. A dynamic periodic nurse routing and scheduling problem with visit time consistency constraints is proposed in Bennett and Erera (2011). A set of dynamic patients needs to be visited according to a given weekly frequency over a given time horizon, and each visit must be scheduled at a time chosen from a fixed menu of allowable appointments. The consistency requirements impose that weekly visits, occurring according to allowable visit-day combinations, must repeat on the same days and times throughout the time horizon. A rolling horizon myopic planning approach for the single nurse case is proposed and tested by exploiting an insertion heuristic. Similarly, a scheduling problem in Home Care where patients arrive dynamically over time and, for the sake of service continuity, they have to be visited at the same day and time each week during their episode of care, is heuristically approached in Demirbilek et al. (2018), Demirbilek et al. (2019). There, the Home Care provider has to decide whether or not to accept the patient and, if accepted, schedule his requests. Again, the case of a single nurse operating in a specific area is addressed. Notice that patient demand is not treated as uncertain in the above mentioned papers. The Home Care system is in fact studied in a dynamic way, from an operating perspective rather than from a planning point of view, and requests have to be managed as soon as patients deterministically enter the system.

On the other hand, Lin et al. (2018) addresses the case where each patient is associated with a list of tasks to be accomplished each week, and he has to indicate a fixed time slot of a certain day of each week within which all his tasks must be executed. Two time slots are considered: a morning and an afternoon one. Therefore, the considered form of consistency can be viewed as a special case of the one addressed in this work, since for each patient all the visits must occur in a unique time slot. Furthermore, this time slot is fixed according to the patient’s indications, rather than to be optimally selected, as in this study.

### Uncertainty plus relaxed forms of consistency

In Sungur et al. (2010) the authors address a courier deliver problem where customer demands and service times are uncertain. Demand and service time uncertainty is not managed via robustness, but via a scenario based approach, by deriving probability estimations starting from some historical scenarios. Neither care continuity nor other forms of temporal consistency are hardly imposed, and so, guaranteed. Rather, the authors introduce the concept of route similarity, and try to generate daily routes which are “similar” to some master plan routes, where similarity is expressed as “the number of customers of the daily route that are within a given distance of any customer on that master plan route”.

### The consistent VRP

In VRP, the focus is progressively moving from company-first strategies to customer-first strategies (Kovacs et al. (2014)), and since customer-satisfaction often increases when service is provided at regular times of the day and by the same driver each time, then consistency now represents a key tool to survive in a competitive market. The Consistent Vehicle Routing Problem, ConVRP, was originally introduced by Groër, Golden and Wasil Groër et al. (2009), who combined arrival time consistency and person-oriented consistency with the traditional VRP constraints. A number of metaheuristics have been then proposed to solve the ConVRP (Kovacs et al. (2014), Tarantilis et al. (2012)) and some generalizations (Kovacs et al. (2015)). The generalized ConVRP was further extended in Kovacs et al. (2015) in a multiobjective perspective. See also Feillet et al. (2014) for an interesting form of time consistency in VRP, where the authors minimize the maximum number of different time slots a patient is visited in.

Notice that, in all the papers cited before, uncertainty issues are not addressed.

## The consistent Robust Home Care problem

### Problem addressed

We are given a set *N* of *n* patients asking for service, a set *O* of skilled caregivers based at a “depot”, a multiple-day planning horizon *W*, and a set *K* of skills, hierarchically organized, so that a caregiver with skill *k* can work requests with skill up to *k*. Each caregiver $\omega \in O$ has a workday length $D_{{\omega }}$, which has to be respected.

Demand uncertainty is addressed, where some patient requests are certain (they will certainly occur), whereas others are uncertain. Each patient *j* is thus associated with both a *certain care plan* $\bar{r}_j$, specifying the number of the certain requests of *j*, for each skill, and an *uncertain care plan* $\tilde{r}_j$, indicating the number of his uncertain requests, again for each skill. Notice that $\tilde{r}_j$ or $\bar{r}_j$ could be missing for a patient *j*: the first case verifies if *j* is a deterministic patient, whereas the latter can be used to denote *j* as a potentially new patient.

Moreover, two forms of *consistency* are considered, along the lines in Kovacs et al. (2014): (*i*) care continuity (or person-oriented consistency) and (*ii*) arrival time consistency. Care continuity bounds to *H* the number of different caregivers who can visit each patient during the time horizon, where *H* is given. Arrival time consistency guarantees that all the visits required by a patient must be performed always in the same daily time slot throughout the planning horizon. Hereafter *T* will denote the set of the time slots in which each day is partitioned, for example morning and afternoon.

The Consistent Robust Home Care Problem consists in (*i*) assigning caregivers to patients by respecting the skill compatibilities, (*ii*) scheduling the patient requests, both certain and uncertain, over the time horizon, (*iii*) determining a routing for each caregiver and each scheduled day, which satisfies the caregiver workday length, while (*iv*) managing demand uncertainty and (*v*) guaranteeing arrival time consistency and person-oriented consistency. The goal to achieve is the caregiver workload balancing. It is thus a challenging problem which extends the Robust Home Care Problem introduced in Cappanera et al. (2018) by managing consistency and demand uncertainty at the same time.

### Modelling issues and assumptions

In order to model the double nature of the patient requests, i.e. either certain or uncertain, each patient $j \in N$ is modelled by a pair of nodes in the logistic network: by a node $j \in \bar{N} = \{1, \ldots , n\}$, representing the certain copy of the patient with the related certain care plan $\bar{r}_j$, and by a node $j+n \in \tilde{N} = \{n+1, \ldots , 2n\}$, representing his uncertain copy with the uncertain care plan $\tilde{r}_j$. The resulting logistic network is thus composed of 2*n* nodes plus the depot.

As in Cappanera et al. (2018), decisions concerning scheduling of skilled (certain and uncertain) visits in the planning horizon, caregiver to patient assignment, and routing of the caregivers are coordinated by means of the concept of * pattern*, introduced in Cappanera and Scutellà (2015), which represents a template for scheduling the patient requests along *W*. More precisely it is assumed that, for each *j*, the requests expressed by the care plan of *j* can be operated according to a set $P_j$ of *a priori* given patterns. Formally, denoting by $P = \cup _{j=1}^{2n}P_j$ the set of all patterns, and assuming that each patient requires at most one visit per day, each pattern $p \in P$ is such that $p(d)=0$ if no service is offered on day *d*, while $p(d)=k$ indicates that a visit of skill *k* is operated according to pattern *p* on day *d*. The pattern $p \in P_j$, selected for *j*, thus determines the scheduling of the requests of *j* along *W*, and it has to be compatible with his care plan, guaranteeing the required number of visits of appropriate skill. Notice that certain or uncertain requests are scheduled depending on whether $j \in \bar{N}$ or $j \in \tilde{N}$.

Demand uncertainty is managed via *sequence-preserving* $\Gamma$-*robustness*, a special case of $\Gamma$-robustness defined in Cappanera et al. (2018) and tailored to vehicle routing problems. $\Gamma$-robustness (Bertsimas and Sim (2004)) allows one to achieve a compromise between degree of robustness and solution cost. In the Robust Home Care context, it addresses scenarios where at most $\Gamma$ uncertain requests (or, equivalently, at most $\Gamma$ nodes from the set $\tilde{N}$) in each tour may realize, where $\Gamma$ is a given parameter. The rationale is that a set of tours, over the planning horizon, which includes all the certain and the uncertain requests would result in a very conservative and unnecessarily expensive solution, since it is likely that only a subset of the uncertain requests will realize. This is why $\Gamma$-robustness focuses on $\Gamma$-tours, i.e. the subtours obtained from a given tour by considering at most $\Gamma$ nodes of $\tilde{N}$, which are said to be generated from that tour. Sequence-preserving $\Gamma$-robustness, instead, considers special $\Gamma$-tours, called sequence-preserving $\Gamma$-tours, i.e. the $\Gamma$-tours where nodes are visited in the same order of the tour that generated it. Besides being computationally tractable, sequence-preserving $\Gamma$-robustness is also greatly appreciated in practice by caregivers, who experiment a limited deterioration of their planned tours in case a disruption occurs.

Sequence-preserving $\Gamma$-robustness is enriched with care continuity (or person-oriented consistency) and arrival time consistency, as previously introduced. Notice that both forms of consistency refer to the original set of patients. In order to handle arrival time consistency, each original caregiver $\omega$ is replaced by |*T*| copies of it, one for each time slot in *T*. The copy of caregiver $\omega$ related to $t \in T$ will be denoted as $\omega _t$. As an example, in case a day is organized in two time slots, i.e. a morning slot and an afternoon slot, each original caregiver $\omega$ is replaced by two copies of it, one relative to the morning shift and the other relative to the afternoon shift. This setting, which is very frequent in practice, at least for palliative cares, is the one our experimentation is based on. Arrival time consistency is thus guaranteed by assigning to each patient caregivers referring to the same time slot in *T*. With a little abuse of the notation, hereafter we shall use *O* to indicate the set of the copies of the caregivers, with $\omega$ denoting its generic element. When necessary, however, $\omega _t$ will be used to specify which copy we are referring to.

According to the definitions given above, a set of daily tours $\mathcal{C}$, including all the certain and the uncertain requests, and an assignment of caregivers to such tours, define a *consistent robust solution* with respect to $\Gamma$ if:

1. for each tour $\tau \in \mathscr{C}$, the length of each sequence-preserving $\Gamma$-tour generated by $\tau$, taking into account the travel time along the links of the tour and the service time at the patients, does not exceed the workday length of the caregiver assigned to $\tau$,
2. the caregivers serving a given patient in the solution, at most *H*, all refer to the same time slot in *T*.

Point 1 guarantees that, for each tour $\tau$ in the solution, the caregiver assigned to $\tau$ is able to perform the tour whatever the (at most $\Gamma$) uncertain requests of $\tau$ will possibly realize, without changing the planned order of the visits. Equivalently, the maximum length of the sequence-preserving $\Gamma$-tours generated by $\tau$ is less than or equal to the workday length of the caregiver. Care continuity and arrival time consistency are ensured by point 2.

Summarizing, the *Consistent Robust Home Care Problem* jointly addresses: (*i*) *care plan scheduling*, assigning a pattern both to the certain copy *j* and to the uncertain copy $j+n$ of each patient *j*, thus scheduling respectively all the certain ($\bar{r}_j$) and the uncertain ($\tilde{r}_j$) requests in the planning horizon; (*ii*) *caregiver assignment*, assigning caregivers to patients while guaranteeing skill compatibility between patient requests and caregivers, and (*iii*) *daily caregiver routing*, determining the tour of each caregiver in each day of the planning horizon, so that the resulting solution constitutes a *consistent robust solution* with respect to $\Gamma$. The goal is to balance the caregiver workload.

We conclude the section by listing the notations used next to state a mathematical model to the problem and to describe the models used to define pattern generation policies.

$$\begin{aligned} \begin{array}{ll} W &{} \text {planning horizon} \\ A &{} \text {set of arcs in the logistic network} \\ O &{} \text {set of skilled caregivers} \\ O_d \subseteq O &{} \text {set of caregivers available on day}\,d,\hbox { for each }d \in W\\ T &{} \text {set of daily time slots} \\ \omega _t &{} \text {copy of caregiver}\,\omega \hbox { for }t \in T \\ K &{} \text {set of skills} \\ s_{\omega } \in K&{} \text {skill of caregiver}\,\omega , \omega \in O \\ D_{{\omega }} &{} \text {workday length of caregiver}\,\omega , \omega \in O\\ \bar{N} = \{1, \ldots , n\} &{} \text {set of certain copies of patients} \\ \tilde{N} = \{n+1, \ldots , 2n\}&{} \text {set of uncertain copies of patients}\\ \bar{r}_{jk} &{} \text {number of certain visits required by}\,j\hbox { in } W\hbox { for skill }k, j \in \bar{N}, k \in K\\ \tilde{r}_{jk} &{} \text {number of uncertain visits required by}\, j \hbox { in }W\hbox { for skill }k, j \in \tilde{N}, k \in K\\ a_{j} &{}\text {service (or assistance) time at patient}\, j, j \in \bar{N} \cup \tilde{N}\\ t_{ij} &{}\text {traveling time from node}\,i\hbox { to node }j, (i,j)\in A\\ H &{}\text {maximum number of caregivers who can visit each patient in}\, W \\ P_{j} &{}\text {set of patterns for}\, j, j \in \bar{N} \cup \tilde{N} \\ P &{}\text {overall set of patterns}. \end{array} \end{aligned}$$

## Mathematical model

Here we present a mathematical formulation of the care continuity constraints and of the arrival time consistency constraints, that is the families of constraints that extend the Robust Home Care Problem in Cappanera et al. (2018) from a consistency perspective. Also the objective function is presented. The complete mathematical formulation is reported in the Appendix.

To formulate the temporal constraints, let us firstly introduce the following families of variables, which model respectively the care plan scheduling, the caregiver assignment and the routing decisions:

$$\begin{aligned}&z_{jp} = \left\{ \begin{array}{ll} 1 \text{ if } \text{ pattern } p \text{ is } \text{ assigned } \text{ to } j \\ 0 \text{ otherwise } \end{array} \right. \quad j \in \bar{N} \cup \tilde{N}, p \in P_j\\&u_{\omega j} = \left\{ \begin{array}{ll} 1 \text{ if } \text{ caregiver } \omega \text{ visits } \text{ patient } j \\ 0 \text{ otherwise } \end{array} \right. \quad \omega \in O, j\in \bar{N} \\&x_{ij}^{\omega d} = \left\{ \begin{array}{lll} 1&{} \text{ if } \text{ caregiver } \omega \text{ travels } \text{ along } (i,j) \\ &{} \text{ on } \text{ day } d \\ 0 &{} \text{ otherwise. } \end{array} \right. \quad (i,j) \in A, d \in W, \omega \in O_d \end{aligned}$$

Notice that, for each caregiver $\omega$, a unique variable $u_{\omega j}$ is introduced for the two nodes, *j* and $j+n$, modelling patient *j*. Furthermore, let us introduce the following variables to guarantee the time consistency:

$$\begin{aligned} Cons_{jt}= & {} \left\{ \begin{array}{ll} 1 \text{ if } \text{ all } \text{ visits } \text{ to } \text{ patient } j \text{ are } \text{ scheduled } \text{ in } \text{ time } \text{ slot } t\\ 0 \text{ otherwise. } \end{array} \right. \quad j\in \bar{N}, t\in T \end{aligned}$$

Using the variables and notation above, the requirement of at most one visit per patient at a day and the *arrival time consistency constraints* can be formulated as follows:

1 $$\sum\limits_{{\omega \in O_{d} }} {\left( {\sum\limits_{{(i,j) \in A}} {x_{{ij}}^{{\omega d}} } + \sum\limits_{{(i,j + n) \in A}} {x_{{i(j + n)}}^{{\omega d}} } } \right)} \le 1\quad \forall j \in \bar{N},\forall d \in W$$

2 $$Cons_{{jt}} \le 1 - \left( {\sum\limits_{{\omega _{{t^{\prime}}} \in O,t^{\prime} \ne t}} {u_{{\omega _{{t^{\prime}}} j}} } /H} \right)\quad \forall j \in \bar{N},t \in T$$

3 $$\begin{aligned}&Cons_{jt} \ge \sum _{\omega _t \in O} u_{\omega _t j}/H\quad \forall j \in \bar{N}, t \in T \end{aligned}$$

Using the routing variables $x_{ij}^{\omega d}$, constraints (1) impose that, on each day and for each patient *j*, at most one of the two nodes modelling the patient, i.e. *j* and $j+n$, can be visited. Constraints (2)-(3) are logical conditions modelling the arrival time consistency requirements. In fact, they guarantee that all the caregivers assigned to a patient refer to the same time slot. Specifically, constraints (2) impose that if at least one caregiver related to a time slot other than *t* is assigned to patient *j*, then variable $Cons_{jt}$ takes value zero and thus patient *j* can not be visited in time slot *t*. Symmetrically, if a caregiver related to a time slot *t* visits patient *j*, then $Cons_{jt}$ is forced to one (see constraints (3)) and a visit to *j* of a caregiver related to a time slot other than *t* is forbidden (see constraints ((2)).

Regarding the *care continuity constraints*, they can be expressed as:

4 $$\begin{aligned}&x_{ij}^{\omega d} \le u_{\omega j} \quad \forall j \in \bar{N}, \forall (i,j) \in A, \forall d \in W, \forall \omega \in O_d \end{aligned}$$

5 $$\begin{aligned}&x_{i,j+n}^{\omega d} \le u_{\omega j} \quad \forall j \in \bar{N}, \forall (i,j+n) \in A, \forall d \in W, \forall \omega \in O_d \end{aligned}$$

6 $$\begin{aligned}&\sum _{\omega \in O} u_{\omega j} \le H \quad \forall j \in \bar{N}. \end{aligned}$$

Together with the consistency constraints (2)-(3), constraints (4)-(6) bound to *H* the number of different caregivers who can visit each patient (in exactly one time slot) during the time horizon.

Finally, a further set of constraints guarantees that the length of each sequence-preserving $\Gamma$-tour does not exceed the workday length of the assigned caregiver. These constraints are given here in a intuitive form and are described in detail in the Appendix:

7 $$\begin{aligned}&R^{\omega d}_{S\Gamma } \le D_{\omega } \quad \forall d \in W, \forall \omega \in O_d \end{aligned}$$

where for each day *d* and each caregiver $\omega \in O_d$, $R^{\omega d}_{S\Gamma }$ represents the maximum length amongst all the sequence-preserving $\Gamma$-tours generated by the tour of $\omega$ on day *d*, say $\tau$. By imposing that such a maximum length cannot exceed the workday duration of $\omega$, we ensure that all the sequence-preserving $\Gamma$-tours generated by $\tau$ satisfy the workday length of $\omega$.

Constraints (7) are used to state the objective function, aiming at balancing the caregiver workloads. This social equity criterion was also investigated in Cappanera and Scutellà (2015), Yalçındağ et al. (2016) and Cappanera et al. (2018). Precisely, we have considered the objective function *minmax*, which minimizes the maximum caregiver utilization factor. In the robust framework under study, the *caregiver utilization factor* is expressed as the total workload of the caregiver during the planning horizon in the worst scenario, over his maximum possible workload. The workload refers to the original set of the caregivers and therefore it must be computed caregiver-wise, i.e. considering the copies of each original caregiver all together. The overall mathematical model can thus be sketched as:

$$\begin{aligned} \begin{array}{lll} \min &{} max_{UF} &{} \\ &{} (1)-(7)\\ &{} \text{ additional } \text{ constraints } \text{ in } \text{ the } \text{ Appendix } \\ &{}\frac{\displaystyle {\sum _{d \in W}^{}} ( \sum _{t \in T} R^{\omega _t d}_{S\Gamma })}{|W| \cdot (\sum _{t \in T} D_{\omega _t})} \le max_{UF}, \forall \text{ original } \text{ caregiver } \omega , \end{array} \end{aligned}$$

where variable $max_{UF}$ bounds from above the maximum caregiver utilization factor, to be minimized.

## The solution approach

We use a pattern based decomposition approach, along the lines preliminarily outlined in Cappanera et al. (2018) and Cappanera and Scutellà (2017). Specifically, for both the certain and the uncertain copy of each original patient, scheduling decisions are fixed within the mathematical model according to the pattern generated for him in a pattern generation phase. In addition, as in Cappanera et al. (2018), each $R^{\omega d}_{S\Gamma }$ expression in constraints (7) is replaced by a suitable upper approximation reported in the Appendix, thus originating a matheuristic approach. Therefore, the pattern generation tool plays a preeminent role in driving efficiently towards good quality solutions. In this study, we propose some pattern generation policies properly designed to take into account the consistency and demand uncertainty aspects which characterize the Consistent Robust Home Care Problem. Specifically, the generated patterns schedule all the requests of a patient *j*, certain and uncertain, in one of the available time slots. In addition, some policies also manage demand uncertainty. Different solution algorithms are thus obtained depending on the specific policy used for generating patterns and, so, for fixing the scheduling decision variables $z_{jp}$. Furthermore, depending on the time slot where the requests of a patient *j* are scheduled, the set of the caregivers who are eligible for *j* in constraints (6) is restricted accordingly. The policies will be described in detail in the next section, whereas in Sect. 6 they will be compared with their consistency-unaware counterparts defined in Cappanera and Scutellà (2017).

### Consistent pattern generation policies

**Fig. 1:**
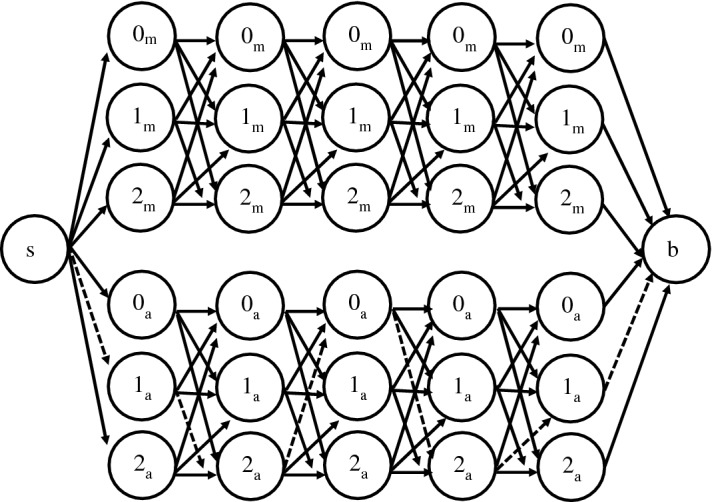
The layered graph $G_W$ for $|W|=5$, $|K|=2$, $|T|=2$, and a pattern example in dashed line

We propose and experiment some alternative pattern generation policies taking into account the consistency requisites and, in some cases, also the demand uncertainty. All the policies involve multicommodity flows on an auxiliary layered network $G_W=(N_W, A_W)$, having one layer for each considered day in the planning horizon *W*, plus a source node (denoted by *s*) and a destination node (denoted by *b*). Each layer in $G_W$ is composed of $|T||K|+|T|$ nodes: |*T*| nodes of type $0_t$, for $t \in T$, which indicate that no visit is scheduled in the time slot *t* of the day corresponding to that layer, and |*T*| nodes for each skill *k* in *K*, denoted by $k_t$, for $t \in T$, which represent the scheduling of a visit of skill *k* in the time slot *t*. Let $L_{d}$ denote the set of nodes in the layer corresponding to day $d \in W$. Then, in $G_W$ there exists a directed arc from *s* to the nodes in the first layer, from each node in the last layer to *b*, and from each node in $L_d$ to each corresponding “consistent” node in $L_{d+1}$ (i.e. each node of type *t* is connected only to nodes of type *t*), for each $d \in W$ but the last, as exemplified in Fig. 1 for a 5-day planning horizon, two skills and two time slots referred to as *m* (morning) and *a* (afternoon).

Any directed *s*-*b* path in $G_W$ thus corresponds to a potential pattern: if the node $k_t$ in $L_d$ is visited, then a visit of skill *k* will occur on day *d* in the time slot *t*; otherwise, i.e. a node of type $0_t$ is visited, then no visits will occur on *d* in the time slot *t*. Notice that, by construction, the nodes composing a path in $G_W$ are all of the same type *t*, i.e. they all refer to the same time slot, so ensuring the time consistency of the scheduled visits. As an example, the path consisting of dashed arcs in Fig. 1, corresponds to pattern (1, 2, 0, 2, 1) according to which exactly four visits will occur in the afternoon: two of skill 1 on Monday and Friday, and two of skill 2 on Tuesday and Thursday respectively.

Four consistency-aware pattern generation policies have been proposed, namely Consistent Spread Uncertainty (*C-SprU*), Consistent Regularize (*C-Reg*), Consistent Balance (*C-Bal*) and Consistent Balance Uncertainty (*C-BalU*). They are based on multicommodity flow models on $G_W$, where a binary commodity is introduced for each $j \in \overline{N} \cup \tilde{N}$ (recall that $\overline{N}$ is the set of nodes corresponding to certain patients, while $\tilde{N}$ is the set of their uncertain copies), and the related flow variables $\{f_{hi}^j\}$ model a directed path, from *s* to *b*, in the layered graph, that is a consistent pattern. Specifically, *C-SprU* pursues the twofold aim of spreading as much as possible the uncertain visits across the planning period so that it is likely that many of them are covered if they realize, while looking for service regularity. This is obtained by minimizing the number of generated consistent patterns for certain patients and maximizing the number of generated consistent patterns for the uncertain ones. In fact, the minimization of the number of different patterns used to schedule certain visits allows to obtain service regularity, while the maximization of the number of different patterns to schedule uncertain visits allows to spread them in the planning horizon. *C-Reg* is the particular case of *C-SprU* arising when all the patients are treated as certain, and therefore it tends to minimize the number of consistent patterns. The last two policies are inspired by balancing criteria. In fact, they take into account the number of visits occurring each day of the planning period, and minimize the difference between the maximum and the minimum such values over *W*. The difference between the two policies concerns the type of visits that are considered: in *C-Bal* all types of visits occurring in a day are considered, i.e. certain and uncertain; in *C-BalU* only uncertain visits are considered.

Notice that the proposed policies generalize the ones used in Cappanera and Scutellà (2015) and the ones described in Cappanera and Scutellà (2017), where consistency requirements are not addressed. In Sect. 6, both the consistency-aware policies and their consistency-unaware counterparts are experimentally investigated. The consistency-unaware policies will be referred to as *SprU*, *Reg*, *Bal* and *BalU*, respectively. The names of the first two policies, namely *SprU* and *Reg*, correspond respectively to *Col* and *FB* in the previous works: their name has been changed to reflect the interesting interpretation they have when consistency is introduced.

The four multicommodity flow models, each relevant to a pattern generator policy, are presented in detail in the following.

#### Spread uncertainty and regularize multicommodity flow models

The pattern generation policy *C-SprU* is based on the following model:

8 $$\begin{aligned} &\text {(C-SprU)}\, \min \sum _{(h,i)\in A_W}(\overline{q}_{hi}- \tilde{q}_{hi}) \nonumber \\&\quad \sum _{(h,i)\in A_W} f_{hi}^j - \sum _{(i,h)\in A_W} f_{ih}^j = \left\{ \begin{array}{ll} -1, &{} \text{ if } i = s,\\ 1, &{} \text{ if } i = b,\\ 0, &{} \text{ otherwise } \end{array}\right. \quad \forall i \in N_W, \forall j \in \overline{N} \cup \tilde{N} \end{aligned}$$

9 $$\begin{aligned}&\sum _{k \in K}\sum _{\begin{array}{c} (s,k_t): \\ k_t \in L_{1} \end{array}}f_{sk_t}^j + f_{s0_t}^j \le \sum _{k \in K}\sum _{\begin{array}{c} (s,k_t): \\ k_t \in L_{1} \end{array}}f_{sk_t}^{j+n} + f_{s0_t}^{j+n} \quad \forall j \in \overline{N}, \forall t \in T\end{aligned}$$

10 $$\sum\limits_{{d \in W}} {\sum\limits_{{t \in T}} { {\sum _{{\begin{array}{*{20}c} {(h,k_{t} ):} \\ {k_{t} \in L_{d} } \\ \end{array} }} f_{{hk_{t} }}^{j} } } } = \bar{r}_{{jk}} \quad \forall j \in \bar{N},\forall k \in K$$

11 $$\sum\limits_{{d \in W}} {\sum\limits_{{t \in T}} { {\sum _{{\begin{array}{*{20}c} {(h,k_{t} ):} \\ {k_{t} \in L_{d} } \\ \end{array} }} f_{{hk_{t} }}^{j} } } } = \tilde{r}_{{jk}} \quad \forall j \in \tilde{N},\forall k \in K$$

12 $$\sum\limits_{{k \in K}} {\sum\limits_{{t \in T}} {\left( {\sum _{{\begin{array}{*{20}c} {(h,k_{t} ):} \\ {k_{t} \in L_{d} } \\ \end{array} }} f_{{hk_{t} }}^{j} + \sum _{{\begin{array}{*{20}c} {(h,k_{t} ):} \\ {k_{t} \in L_{d} } \\ \end{array} }} f_{{hk_{t} }}^{{j + n}} } \right)} } \le 1\quad \forall j \in \bar{N},\forall d \in W$$

13 $$\sum\limits_{{j \in \bar{N} \cup \tilde{N}}} {a_{j} } \sum\limits_{{k^{\prime } \ge k}} { {\sum _{{\begin{array}{*{20}c} {(h,k_{t}^{\prime } ):} \\ {k_{t}^{\prime } \in L_{d} } \\ \end{array} }} f_{{hk_{t}^{\prime } }}^{j} } } \le \epsilon \sum _{{\begin{array}{*{20}c} {\omega _{t} \in O_{d} :} \\ {s_{{\omega _{t} }} \ge k} \\ \end{array} }} D_{{\omega _{t} }} \quad \forall d \in W,\forall k \in K,\forall t \in T$$

14 $$\begin{aligned}&\sum _{j \in \overline{N}} f_{hi}^j \le |\overline{N}| \cdot \overline{q}_{hi}\quad \forall (h,i)\in A_W \end{aligned}$$

15 $$\begin{aligned}&\sum _{j \in \tilde{N}} f_{hi}^j \le |\tilde{N}| \cdot \tilde{q}_{hi}\quad \forall (h,i)\in A_W \end{aligned}$$

16 $$\begin{aligned}&f_{hi}^j \in \{0,1\}\quad \forall (h,i)\in A_W, \forall j \in \overline{N} \cup \tilde{N} \end{aligned}$$

17 $$\begin{aligned}&\overline{q}_{hi} \in \{0,1\}\quad \forall (h,i)\in A_W \end{aligned}$$

18 $$\begin{aligned}&\tilde{q}_{hi} \in \{0,1\}\quad \forall (h,i)\in A_W. \end{aligned}$$

Constraints (8) are the classical commodity-wise flow conservation constraints, which are used to design a path, i.e. a pattern, for each copy (certain and uncertain) of each patient, in order to schedule his certain and uncertain requests, respectively. As observed, by construction the nodes in a path all refer to the same time slot, so ensuring the visit consistency for each $\forall j \in \overline{N} \cup \tilde{N}$. But, as previously remarked, the consistency must be guaranteed for the original set of the patients, and not separately for their certain and uncertain copies. Therefore, constraints (9) are introduced to guarantee the consistency of all visits of a patient throughout the planning horizon. In fact, if the pattern designed for the uncertain copy of a patient does not comprise nodes related to a time slot *t*, then no nodes referring to *t* can belong to the pattern of his certain copy, for each time slot *t*.

Flow variables are also used to guarantee the proper number of visits for each skill thanks to constraints (10) and (11), and therefore model a pattern which is compatible with the patient care plan, both for the certain (10) and the uncertain (11) requests of each patient. Furthermore, they allow to guarantee that at most one visit per day will be scheduled for each patient (12). Constraints (13) take into account, skill by skill, the operators’ availability on each daily time slot. In fact they impose, separately for skill *k* and each day, that the sum of the service time ($a_{j}$) of the visits scheduled in each time slot of the day does not exceed the related availability of the operators of skill at least *k*, properly reduced by a parameter $\epsilon$. Parameter $\epsilon$ reduces the caregiver availability thus preventing infeasibilities that might occur when routing is considered: in fact, pattern generation neglects the traveling times. The auxiliary variables $\overline{q}_{hi}$ and $\tilde{q}_{hi}$ are introduced to detect which arcs are used to design consistent patterns: specifically, for a given arc (*h*, *i*), $\overline{q}_{hi}$ will be set to one if the arc is used by a patient in $\overline{N}$, while $\tilde{q}_{hi}$ will be set to one when (*h*, *i*) is used by a patient in $\tilde{N}$. Finally, constraints (14) and (15) link together the flow variables $f_{hi}^j$ respectively with the design variables $\overline{q}_{hi}$ and $\tilde{q}_{hi}$, and they guarantee that if arc (*h*, *i*) is used, i.e. $\overline{q}_{hi}=1$ (respectively $\tilde{q}_{hi}=1$), it can be crossed by any number of certain (uncertain) patients, whereas if that arc is not used, i.e. $\overline{q}_{hi}=0$ (respectively $\tilde{q}_{hi}=0$), then it cannot be traversed. By minimizing the difference between the total number of arcs used for certain patients and the total number of arcs used for uncertain copies, the model will tend to regularize service and spread uncertainty, as previously observed.

*C-Reg* is the particular case of *C-SprU* where all patients are treated as certain. The total number of arcs used is so minimized, pursuing service regularity.

#### Balanced multicommodity flow models

The two additional models, based on a balancing criterion, consider the number of visits occurring each day, and minimize the difference between the maximum and the minimum of such values over *W*. This is done by means of two variables, namely, $z_{\text {min}}$ and $z_{\text {max}}$, which represent respectively the minimum and the maximum number of visits occurring daily in *W*. The difference between the two models concerns the type of visits considered: in *C-Bal* both certain and uncertain visits occurring in a day are considered, whereas in *C-BalU* only uncertain visits are taken into account. The first of the two models is:

19 $$\begin{aligned}&{\text{(C - Bal)}}\quad \min \quad z_{{{\text{max}}}} - z_{{{\text{min}}}} \\ &{\text{s.t.}}(8) - (13)\\&z_{{{\text{min}}}} \le \sum\limits_{{j \in \bar{N} \cup \tilde{N}}} {\sum\limits_{{k \in K}} {{\sum\limits_{{t \in T}} {\sum _{{\begin{array}{*{20}c} {(h,k_{t} ) \in A_{w} } \\ {{\text{s.t.}}k_{t} \in L_{d} } \\ \end{array} }} } f_{{hk_{t} }}^{j} } } } \le z_{{{\text{max}}}} \quad \forall d \in W\\&f_{{hi}}^{j} \in \{ 0,1\} ,\forall (h,i) \in A_{W} ,\forall j \in \bar{N} \cup \tilde{N}.\end{aligned}$$

As in the previous models, the block of constraints (8)-(13) is related to the flow variables $\{f_{hi}^j\}$, $\forall j \in \overline{N} \cup \tilde{N}$. Then in (19), for each day, the total number of visits performed on that day, independently of the skill, is bounded between the lower bound $z_{\text {min}}$ and the upper bound $z_{\text {max}}$. The difference between such bounds is minimized in an attempt to spread the visits equally among the days.

The model *C-BalU* is exactly as *C-Bal* but for constraints (19), which are replaced by the following counterpart, where the daily number of visits is computed by considering only uncertain visits:

20 $$\begin{aligned}&\text {(C-BalU)} \quad z_{\text {min}} \le \sum _{j \in \tilde{N}} \sum _{k \in K} \sum _{t \in T} \sum _{\begin{array}{c} (h,k_t) \in A_w \\ \text {s.t. } k_t \in L_d \end{array}} f_{hk_t}^j \le z_{\text {max}} \quad \forall d \in W. \end{aligned}$$

## Computational experiments

In this section, we first describe the instances of our experimental setting, which are characterized by $|T|=2$ time slots: morning and afternoon. Then we show the results obtained when the policies in Sects. 5.1.1 and 5.1.2, both the consistency-aware and their consistency-unaware counterparts, are used within the pattern based decomposition approach, giving rise to alternative algorithms to the Consistent Robust Home Care problem. The following metrics are used to evaluate the impact of the alternative policies: (*i*) number of possible realizations of the uncertain visits; (*ii*) workload balance among caregivers, and (*iii*) optimality gap of the solution obtained. The number of possible realizations of uncertain visits is determined by looking at the solution of the optimization model. Specifically, for each duplicated caregiver and for each day in the planning horizon, we store the minimum between the number of uncertain nodes present in the tour of the caregiver and $\Gamma$. Such a value represents the maximum number of uncertain visits that can be covered in that tour if they realize. The sum of these values over all the tours and the days is the number of possible realizations of the uncertain visits. Instead, the maximum theoretical number of uncertain visits that can be realized by our approach can be computed a priori and is given by the minimum between the number of uncertain visits in the instance and $2 \cdot \Gamma \cdot |W| \cdot |O|$, where $2 \cdot |O|$ is the number of (duplicated) caregivers, |*W*| the planning horizon length and $\Gamma$ the uncertainty parameter. The workload balance is measured as the percentage difference between the maximum and the minimum caregiver utilization factor and it is referred to as $\Delta _{UF}$. The first two metrics refer to solution quality whereas the third one refers to computational efficiency. In the following, for each group of instances, we report separately the results relative to solution quality and to efficiency. A discussion of the main insights obtained concludes the section.

### The test bed

We generated 33 consistent robust instances by extending the data set in Cappanera and Scutellà (2015), Yalçındağ et al. (2016) and Cappanera and Scutellà (2017), which is publicly available at http://www.di.unipi.it/optimize/. The original data set comes from a real public provider operating in the North of Italy. The data set used in these experiments comprises 9 instances generated by original instances with 60 patients on a 5-day planning horizon, and 24 instances coming from original instances with 100 patients on a 6-day horizon. Each instance is characterized by 2 types of hierarchical skills, 3 operators, travelling times obtained via Google Maps, a service time of 45 minutes for each visit, and $\Gamma =1$. The percentage of uncertain visits is equal to 20% of all the visits for instances with 60 patients and 20% or 30% for instances with 100 patients (12 instances with 20% and 12 with 30%). Instance names contain information on the number of patients in the original instance (either 60 or 100), part of the name of the corresponding original instance (as an example, 0-1 refers to instance 1 in group 0), and the fraction of certain visits (either 0.7 or 0.8). Specifically, consistent robust instances are generated from the nominal ones as follows. For a given nominal instance, patients are ordered according to the total number of visits they require regardless of the skill. Then, starting from the patients with the highest number of visits, at each iteration a patient is randomly selected and part of his requests (a number randomly generated) are classified as uncertain and assigned to a new patient. The generator guarantees that, for each skill, uncertain visits are proportional to the number of visits of that skill in the original instance. Notice that patients are either certain or uncertain as a result of the procedure above. Also note that due to the procedure used to generate the robust instances, the number of patients in the robust instance is greater than in the original one while the number of visits is the same. Detailed information on the instances is given in Table 1, where average information is also reported for each group of instances.

The experiments, consisting in solving the pattern based matheuristic under the pattern generation policies presented in Sect. 5, have been performed on an Intel(R) Core(TM) i7-4770 CPU @ 3.40GHz with 4 processors using Cplex 12.6, by imposing a time limit (2 hours) and a memory limit (2 Gbyte). The impact of extending such limits has been evaluated for the biggest instances in the test bed, i.e. for a subset of the instances with 100 patients.

**Table 1:** Test bed description: TV is the number of Total Visits—UV is the number of Uncertain Visits

Instance	Patients	TV	UV	UV/TV(%)
60-0-0-0.8	69	81	17	20.99
60-0-1-0.8	70	81	19	23.46
60-0-2-0.8	70	81	19	23.46
60-1-0-0.8	67	76	11	14.47
60-1-1-0.8	67	76	10	13.16
60-1-2-0.8	67	76	11	14.47
60-2-0-0.8	70	79	16	20.25
60-2-1-0.8	70	79	16	20.25
60-1-2-0.8	70	79	16	20.25
Average	68.89	78.67	15.00	18.97
100-0-0-0.8	121	161	33	20.50
100-0-1-0.8	121	161	33	20.50
100-0-2-0.8	121	161	33	20.50
100-1-0-0.8	117	170	35	20.59
100-1-1-0.8	117	170	35	20.59
100-1-2-0.8	117	170	35	20.59
100-2-0-0.8	117	163	33	20.25
100-2-1-0.8	117	163	33	20.25
100-2-2-0.8	117	163	33	20.25
100-3-0-0.8	112	162	33	20.37
100-3-1-0.8	112	162	33	20.37
100-3-2-0.8	112	162	33	20.37
Average	116.75	164.00	33.50	20.43
100-0-0-0.7	130	161	45	27.95
100-0-1-0.7	130	161	45	27.95
100-0-2-0.7	130	161	45	27.95
100-1-0-0.7	130	170	52	30.59
100-1-1-0.7	130	170	52	30.59
100-1-2-0.7	130	170	52	30.59
100-2-0-0.7	131	163	50	30.67
100-2-1-0.7	131	163	50	30.67
100-2-2-0.7	131	163	50	30.67
100-3-0-0.7	123	162	47	29.01
100-3-1-0.7	123	162	47	29.01
100-3-2-0.7	123	162	47	29.01
Average	128.50	164.00	48.50	29.56

### The 60-patient instances

In this section, we report separately computational results on consistency-unaware policies and on the consistency-aware ones respectively in Sects. 6.2.1 and 6.2.2. A comparison between the two kinds of policies concludes the section (Sect. 6.2.3).

#### Consistency-unaware policies

**Fig. 2:**
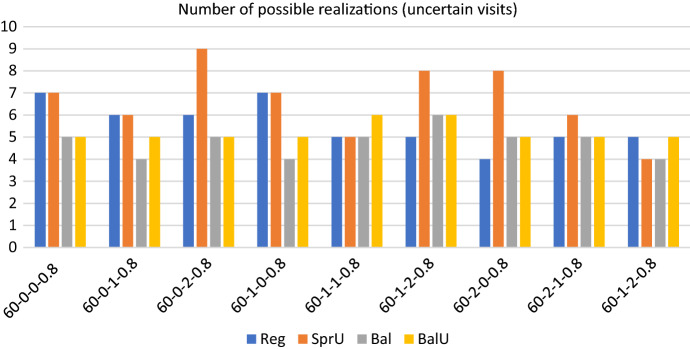
Realizations of uncertain visits: 60 patients - consistency-unaware

**Fig. 3:**
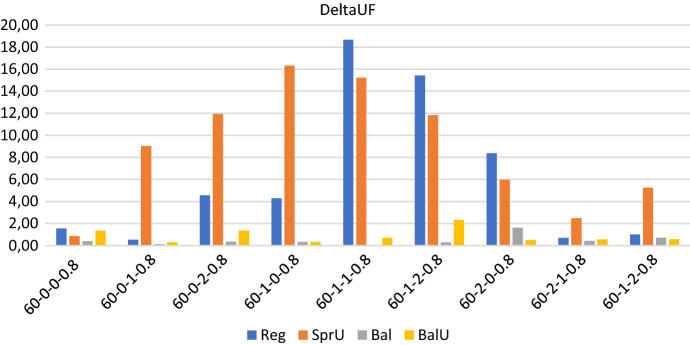
Operator utilization factor: 60 patients - consistency-unaware

**Fig. 4:**
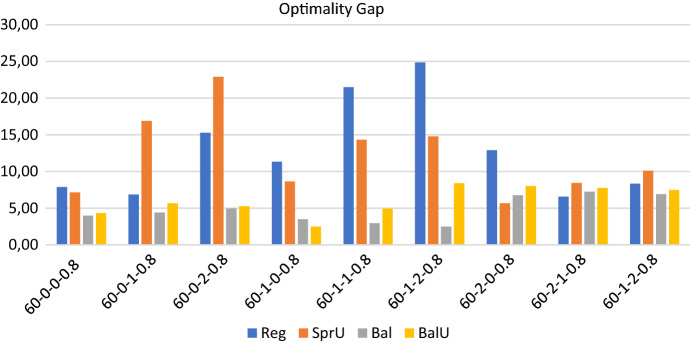
Optimality gap: 60 patients - consistency-unaware

Figures 2, 3 and 4 show respectively the number of possible realizations of the uncertain visits, the measure of the workload balance $\Delta _{UF}$, and the optimality gap for the four consistency-unaware pattern generation policies described in Sects. 5.1.1 and 5.1.2, i.e. *SprU*, *Reg*, *Bal*, and *BalU*.

*Solution quality* On this set of instances, the average maximum theoretical number of uncertain visits that can be realized is 15, and Fig. 2 clearly shows that *SprU* is particularly effective in scheduling the uncertain visits giving almost everywhere the maximum number of uncertainty realizations among the four policies. By comparing the two policies taking into account a balancing criterion, i.e. *Bal* and *BalU*, we can observe that generally, incorporating uncertainty in designing pattern, i.e. using *BalU*, is a plus.

In regards to the workload distribution among the caregivers, Fig. 3 reveals that, as expected, the two policies based on a balancing criterion allow to better control the workload unbalance among the caregivers and to obtain very small values for $\Delta _{UF}$. On the contrary, the policies *Reg* and *SprU* may generate solutions with an unsatisfying value of $\Delta _{UF}$, which reaches the maximum value of 18.67 when policy *Reg* is used.

*Computational efficiency* In regards to computational efficiency, Fig. 4 shows that, as for the workload balance, the policies based on a balancing criterion give better results.

As an overview, we report some aggregate information on computational efficiency: regardless of the policy adopted, the average computational time is 3173.45 seconds and the average optimality gap is 8.94 with a peak of 24.85 on one instance. On the 36 runs obtained solving the 4 policies on the 9 instances, we report that 30 runs terminate for memory limit and 6 for time limit. Thus, the optimality of the solution is never certified on this set of experiments. The prevalence of terminations due to memory limit over those due to time limit (30 over 6) reveals that the enumeration tree in the branch and bound algorithm grows really fast. This fact thus highlights that the efficiency of the algorithm would not benefit from an increase of computational time. Indeed, as it will be shown for a subset of the bigger instances in the test bed, the performance of the algorithm does not seem to benefit from either an increase in time or memory. Rather, the definition of ad hoc methods that allow to control the explosion of memory represents an interesting line of future research.

#### Consistency-aware policies

**Fig. 5:**
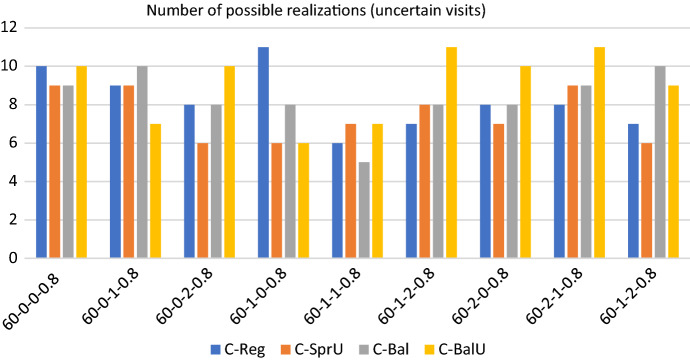
Realizations of uncertain visits: 60 patients - consistency-aware

**Fig. 6:**
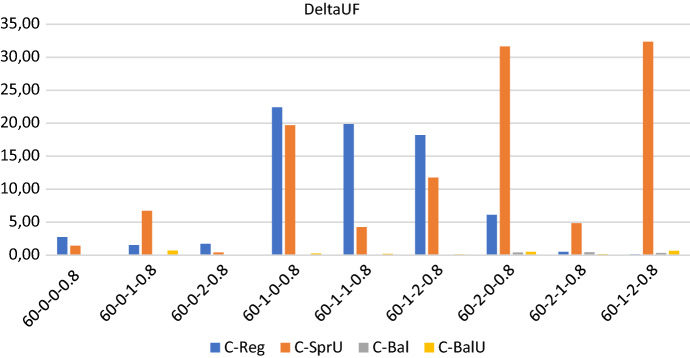
Operator utilization factor: 60 patients—consistency-aware

**Fig. 7:**
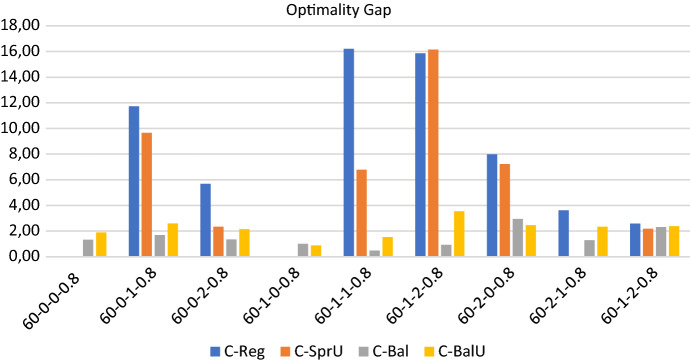
Optimality gap: 60 patients—consistency-aware

We now evaluate the impact of introducing consistency in pattern generation. Similarly to what done for the consistency-unaware policies, Figs. 5, 6 and 7 show respectively the number of uncertainty realizations, the measure of the workload balance $\Delta _{UF}$, and the optimality gap for the four consistency-aware pattern generation policies described in Sects. 5.1.1 and 5.1.2, i.e. *C-Reg*, *C-SprU*, *C-Bal*, and *C-BalU*.

*Solution quality* All of the policies seem to be quite effective in scheduling uncertain visits, with policies *C-Reg* or *C-BalU* giving better results than the others but on two instances where the best performance is obtained by *C-Bal*. As for the consistency-unaware policies, the best results in terms of workload balance are given by the policies which incorporate a balancing criterion, as Figs. 6 clearly reveals.

*Computational efficiency* Similarly, the best results in terms of computational efficiency are given by the policies guided by a balancing criterion, see Fig. 7.

#### Consistency-unaware vs consistency-aware policies

**Fig. 8:**
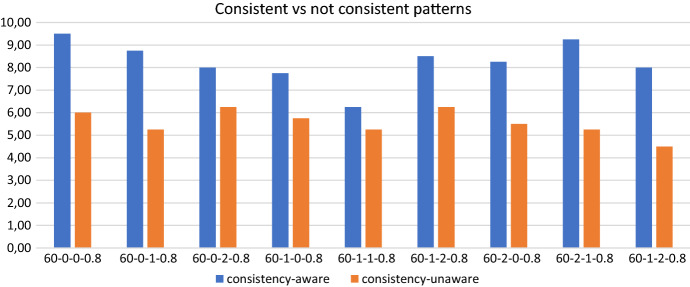
Consistency vs not consistency in pattern generation for 60 patients: impact on the number of possible realizations

The direct comparison between the consistency-unaware policies and the consistency-aware ones, shows that the latter policies allow to satisfy a greater number of uncertain visits almost everywhere, as reported in Fig. 8. In addition, introducing consistency allows to obtain a considerable improvement also from the computational efficiency point of view. Indeed, as an overview of aggregate information, for the consistency-aware policies we have that the average computational time is 3199.23 seconds, and the average optimality gap is decreased to 3.92 when compared with the 8.94 value obtained on average with the consistency-unaware policies. On the 36 runs, we report that 23 runs terminate for memory limit, 8 terminate for time limit and 5 are solved to optimality. Importantly, the number of runs prematurely interrupted due to the memory limit diminishes remarkably from 30 to 23, even if this implies a slight increase of the average computational time.

Summarizing, we can conclude that on the 60-patient instances, all of the policies, i.e. both the consistency-aware and the consistency-unaware ones, are able to find solutions of good quality to the Consistent Robust Home Care problem. However, introducing consistency allows to satisfy a greater number of uncertain visits with respect to the consistency-unaware counterparts and to gain in computational efficiency.

### The 100-patient instances

In this section, we report separately computational results obtained on instances characterized by a percentage of uncertain visits equal to 20% (Sect. 6.3.1) and 30% (Sect. 6.3.2) respectively.

Remarkably, we observe that on the 100-patient instances the consistency-aware policies play a preeminent role in gaining feasibility. Indeed, computational results obtained on the instances with a percentage of uncertain visits equal to 20%, show that the consistency-unaware policies are able to find a feasible solution within the memory and time limits imposed only on 12 over 48 runs, where the number of runs is given by the number of policies (4) times the number of instances (12). Thus, they are able to determine a feasible solution in 25% of the cases. On the contrary, the consistency-aware policies always determine a feasible solution, independently of the specific policy adopted. For this reason, in the following, we only report computational results for the consistency-aware policies.

#### The 100-0.8 instances

**Fig. 9:**
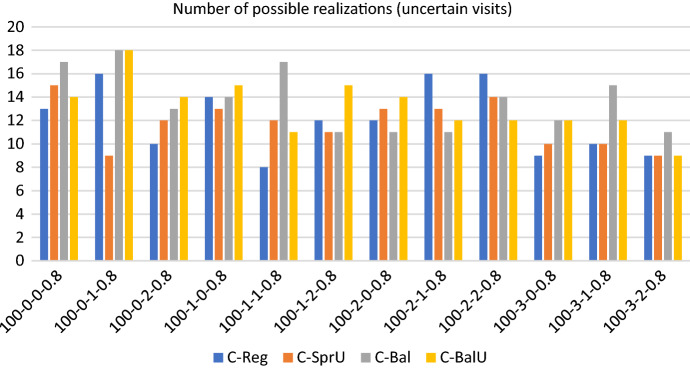
Realizations of uncertain visits: 100 patients, 20% uncertain visits—consistency-aware

**Fig. 10:**
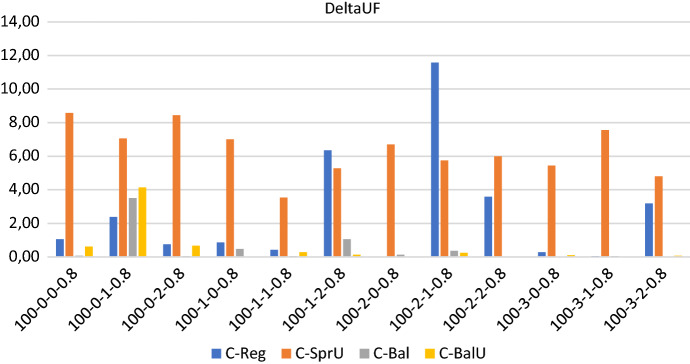
Operator utilization factor: 100 patients, 20% uncertain visits—consistency-aware

**Fig. 11:**
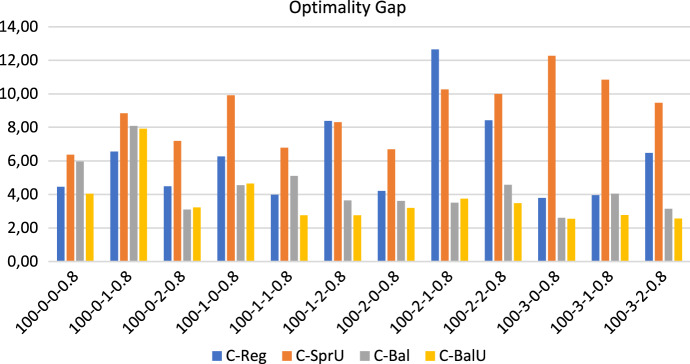
Optimality gap: 100 patients, 20% uncertain visits - consistency-aware

Figures 9, 10 and 11 show respectively the number of uncertainty realizations, the measure of the workload balance $\Delta _{UF}$, and the optimality gap for the consistency-aware pattern generation policies described in 5.1.1 and 5.1.2, i.e. *C-Reg*, *C-SprU*, *C-Bal*, and *C-BalU*.

*Solution quality* On this set of instances, the average maximum theoretical number of uncertain visits that can be realized is 33, and Fig. 9 shows that the two policies driven by a balancing criterion, i.e. *C-Bal*, and *C-BalU*, are well able to satisfy the greatest number of uncertain visits. *C-Bal* and *C-BalU* are also effective in containing the workload imbalance, as Fig. 10 shows.

*Computational efficiency* As shown by Fig. 11, *C-Bal* and *C-BalU* can also be solved more efficiently than the others.

As an overview of aggregate information, we have that the average computational time is 4478.11 seconds and the average optimality gap is 5.75 with a peak of 12.65 obtained by policy *C-Reg* on one instance. On the 48 runs, we report that 30 runs terminate for memory limit and 18 terminate for time limit.

**Fig. 12:**
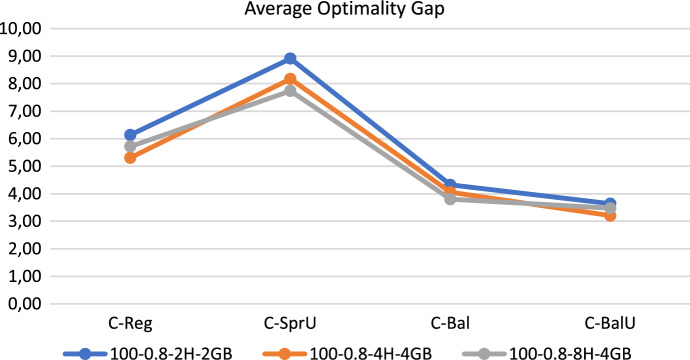
Effects of extending time and memory limits on efficiency

To better understand the impact of imposing time and memory limits on the performance of the algorithm, we run an additional set of tests. Since for both the 60-patient instances and the 100-0.8 ones the terminations due to memory limit prevail over those due to time limit, increasing the computational time alone would not lead to improvements. For this reason we increased both limits at the same time. We have therefore run again the algorithm on the 100-0.8 instances progressively increasing the time to 4 and 8 hours and the memory to 4 Gbyte. Figure 12 shows, for each policy, the average gap obtained on the 12 instances of the group 100-0.8 for each combination of values used as time and memory limits. The figure clearly shows that the computational efficiency of the algorithm does not improve significantly as time and memory availability increases. Setting the time limit to 2 hours and the memory limit to 2 Gbyte seems therefore a reasonable choice and this setting of the parameters will be used also for the following experiments.

#### The 100-0.7 instances

**Fig. 13:**
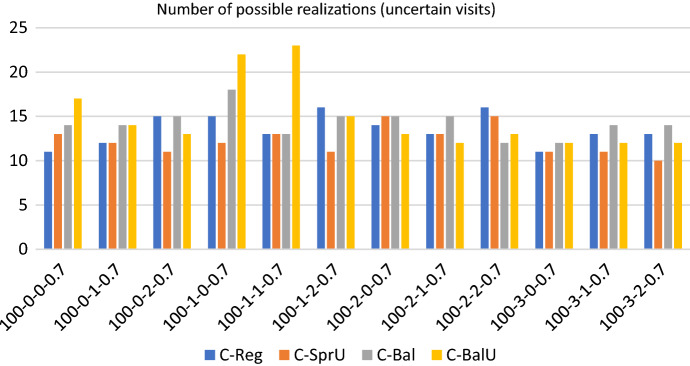
Realizations of uncertain visits: 100 patients, 30% uncertain visits - consistency-aware

**Fig. 14:**
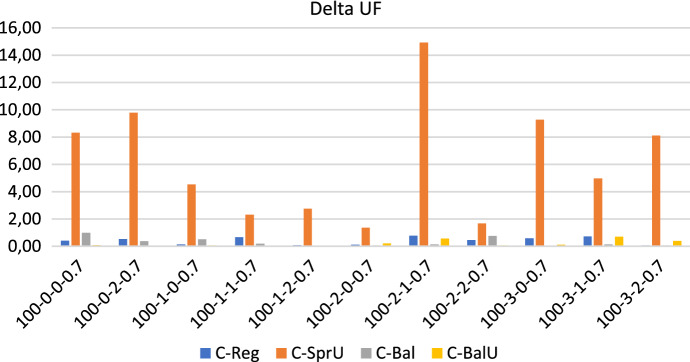
Operator utilization factor: 100 patients, 30% uncertain visits - consistency-aware

**Fig. 15:**
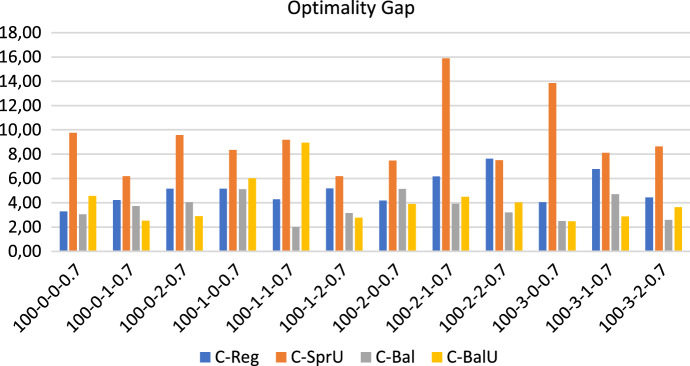
Optimality gap: 100 patients, 30% uncertain visits—consistency-aware

Figures 13, 14 and 15 show respectively the number of uncertainty realizations, the measure of the workload balance $\Delta _{UF}$, and the optimality gap for the consistency-aware pattern generation policies *C-Reg*, *C-SprU*, *C-Bal*, and *C-BalU*.

*Solution quality* All of the policies seem to be quite effective in covering uncertain visits, with policy *C-BalU* usually obtaining the highest number of possible realizations. In regards to the workload balance, all of the policies are very effective in guaranteeing an equal distribution of the workload among the caregivers with the only exception of policy *C-SprU*, which gets a $\Delta _{UF}$ worse than that obtained by the other policies and in one case exhibits a workload unbalance greater that 14%.

*Computational efficiency* The optimality gap is very low for all the policies except *C-SprU*, which obtains the worst performance.

As an overview, we report some aggregate information on computational efficiency: regardless of the policy adopted, the average computational time is 4156.33 seconds and the average optimality gap is 5.49. On the 36 runs obtained solving the 4 policies on the 9 instances, we report that 33 runs terminate for memory limit and 15 for time limit.

Interestingly, when compared with the 100-0.8 instances, the performances obtained on the 100-0.7 instances seem to be more stable across the four policies that show quite similar behaviours.

### Results in brief

**Fig. 16:**
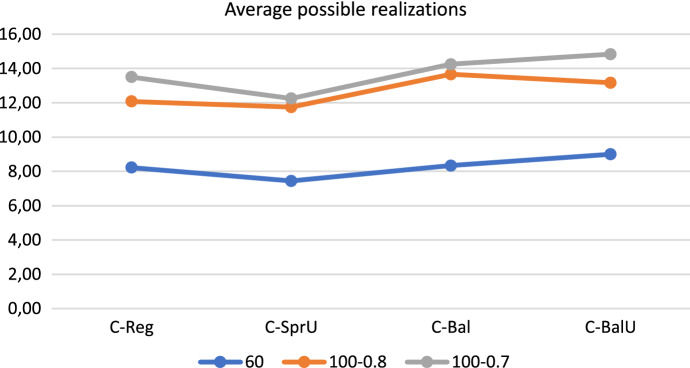
Realizations of uncertain visits: average results—consistency-aware

**Fig. 17:**
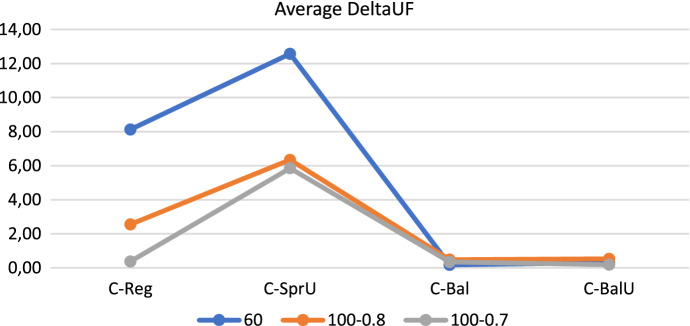
Operator utilization factor: average results—consistency-aware

**Fig. 18:**
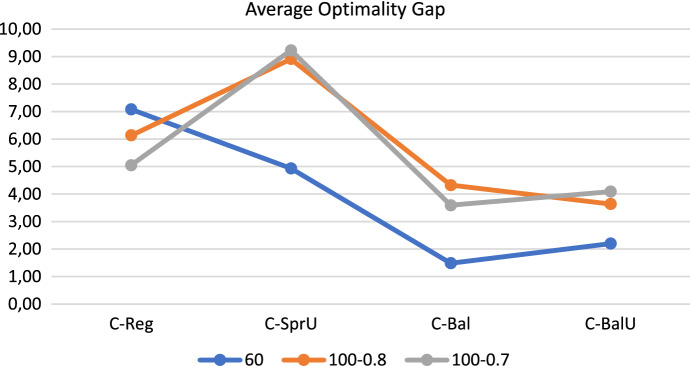
Optimality gap: average results—consistency-aware

In order to provide an overview of the results reported so far, we give in this section average results obtained with the four consistency-aware policies on the three groups of instances. Specifically, Figs. 16, 17, and 18 show respectively the average number of uncertainty realizations, the average measure of the workload balance $\Delta _{UF}$, and the average optimality gap. These aggregate results allow to confirm the trend discussed separately for each group of instances, i.e.: (*i*) all the policies are effective in covering possible realizations of uncertain visits; (*ii*) as desirable, the two policies incorporating balancing issues (*C-Bal* and *C-BalU*) are well able to provide very good value of $\Delta _{UF}$, and also (*iii*) limited optimality gaps.

We conclude the section with a brief discussion of the main achievements obtained. The computational results show that the decomposition approach proposed in this paper, at least on the tested set of instances, is a viable tool to successfully address the challenging Consistent Robust Home Care problem. In fact, the four consistency-aware policies are able to achieve good quality solutions. In particular: (*i*) introducing consistency in pattern generation policies is an effective tool both to increase the number of uncertainty realizations (see the 60-patient instances) and to increase remarkably the number of instances for which a feasible solution is found (see the 100-patient instances); (*ii*) incorporating a balancing criterion in pattern generation policies positively impacts both on reducing the workload imbalance among the caregivers - and this is a quite expected result, and to increase computational efficiency - and this is a less expected result. Indeed, the pattern generation policies that consider the workload balance seem to take the “right” scheduling decisions in the first phase of the decomposition approach, especially when taking into account also consistency and uncertainty; the resulting model solved in the second phase is thus guided efficiently towards good quality solutions. The algorithm based on patterns generated via the policy *C-BalU* seems thus to be particularly appealing in computing consistent solutions also characterized by a high degree of robustness and a very good workload balance. Such an algorithm could be a valuable tool for providers interested in efficiently addressing uncertainty and pursuing consistency of the patient schedules while balancing the caregiver workload, so achieving a good trade-off between patient-centered and caregiver-centered perspectives. The value of consistency on the patient perspective will be better emphasized next.

## The value of consistency on patient perspective

In this section, we report computational results showing the importance of considering consistency in pursuing a patient-centered perspective. Specifically, we solved two groups of instances, both the 60-patient and the 100-0.8 instances, without imposing any kind of consistency, and we measured the average (over all the instances in a group) percentage of patients for whom a violation of care continuity (%CC violation in Table 2) or time consistency (%TC violation in Table 2) occurs. When care continuity is disregarded, constraints (6) are relaxed, thus allowing that a patient is visited each time by a different caregiver. When time consistency is neglected, the pattern associated with each patient in the first phase comes without any information on the session (morning or afternoon) in which visits will occur.

More in detail, to measure the value of adding consistency to the model, we run a set of additional experiments in which we disregard both types of consistency at the same time as described above. Starting from the same patterns and under the same time and memory limits, the quality of the solutions obtained when consistency is neglected is evaluated against solution quality when consistency is addressed. In both cases, the four consistency-aware policies are used in the first phase to generate patterns.

Table 2, separately for each group of instances, reports the average percentage of patients for whom a violation of care continuity occurs (column %CC violation), the average percentage of patients for whom a violation of time consistency occurs (column %TC violation), the total number of instances for which a feasible solution is obtained within the time and memory limits imposed (column Solved), and finally, in the last four columns, the same type of information on the capability of a policy to provide feasible solutions, separately for each policy.

The computational results show that not considering time consistency has a greater (negative) impact on solution quality than not considering care continuity. Specifically, about the 30% of patients with more than one visit is visited by more than one operator. Thus, not assuring person-oriented consistency compromises solution quality from the patient perspective, but not addressing time consistency deteriorates solution quality even more. In fact, about the 60% of patients with more than one visit is visited both in the morning and in the afternoon during the planning horizon. Interestingly, the two groups of instances exhibit similar trends. The two groups of instances, instead, are quite different in terms of capability of the policies to find feasible solutions. While on the 60-patient instances a feasible solution is found for each of the 9 instances and for each of the four alternative policies (36 runs), on the 100-0.8 instances only 11 over 48 (12 instances and 4 policies) runs led to feasible solutions. On such instances, policy *C-Reg* seems to be more effective than the others obtaining the maximum number of successful runs. Note that, when consistency is assured, both for the 60-patient instances and for the 100-patient ones, all of the four alternative policies always determine a feasible solution.

We can thus conclude that addressing consistency has undoubtedly a beneficial effect on the solution quality from a patient-centered perspective. In addition, it also affects positively the computational efficiency. This could appear quite surprising, but is indeed in accordance with what shown in Sect. 6. Even if in the decomposition approach proposed to solve the problem the information on the session is fixed, in fact, the approach is able to find feasible solutions in a greater number of runs with respect to the case in which consistency is not addressed, thus confirming that the consistency-aware policies are effective in guiding the search towards good quality solutions.

**Table 2:** Impact of consistency on solution quality

Instance Group	%CC violation	%TC violation	Solved	Solved (policy-wise)			
				*C-Reg*,	*C-SprU*,	*C-Bal*,	*C-BalU*
60	31.79	62.07	36/36	9	9	9	9
100-0.8	34.94	59.70	11/48	5	1	2	3

## Conclusions

We have introduced and studied a new problem in Home Care, i.e. the Consistent Robust Home Care problem, where arrival time consistency, person-oriented consistency and demand uncertainty are addressed while jointly optimizing assignment, scheduling and routing decisions over a multiple-day time horizon.

We have presented a mathematical model to formulate the problem and a pattern based algorithmic framework to solve it. The framework is obtained from the proposed mathematical model via decomposition, i.e. suitably fixing the scheduling decisions through the concept of pattern. Some alternative pattern generation policies, based on multicommodity flow formulations on an auxiliary graph, and explicitly taking into account the consistency requirements in addition to the demand uncertainty, have been proposed as a tool to derive alternative algorithmic approaches from the general pattern-based framework. The wide computational experience has shown that some of the proposed consistent algorithms are able to compute very good quality solutions, both in terms of robustness and in terms of balancing of the caregiver workload. This verifies mainly when using pattern generation policies which explicitly take into account consistency, uncertainty and balancing issues. In addition, the proposed approaches are computationally efficient, by considering the hardness of the Home Care aspects which are jointly optimized. Also, a comparison with an approach in which arrival time consistency and person-oriented consistency are not imposed, shows that considering consistency has a beneficial effect on the solution quality from a patient-centered perspective, with a positive impact on the efficiency of the approach.

We plan to computationally compare our approach, which realizes the arrival time consistency by selecting a daily time slot per patient during the optimization process, with a less flexible approach based on time windows, by investigating the impact of the number of considered time slots on efficiency and flexibility. Furthermore, we plan to further extend the studied Home Care Problem by incorporating other kinds of constraints reflecting a patient-centered perspective, and considering also decisions relative to special equipments or devices (Cappanera et al. (2020)) which might be required to service patients.

We believe that the proposed algorithm, suitably integrated with IoT tools, can be the engine of a decision support system to assist Home Care service providers. The importance of such tools is more evident today than ever: an optimized management of Home Care services is essential both in the emergency, to manage the exponential growth in demand due to the SARS-COV-2 pandemic we are experiencing, and in the long term to increase the quality of service according to a patient-centered perspective.

## Appendix: the complete mathematical model

Here we present the complete mathematical formulation to the Consistent Robust Home Care Problem. The model is based on the following notation:

$$\begin{aligned} \begin{array}{ll} W &{} \text {planning horizon} \\ A &{} \text {set of arcs in the logistic network} \\ O &{} \text {set of skilled caregivers} \\ O_d \subseteq O &{} \text {set of caregivers available on day}\,d,\hbox { for each }d \in W\\ T &{} \text {daily time slots} \\ \omega _t &{} \text {copy of caregiver}\,\omega \hbox { for }t \in T \\ K &{} \text {set of skills} \\ s_{\omega } \in K&{} \text {skill of caregiver}\,\omega , \omega \in O \\ D_{{\omega }} &{} \text {workday length of caregiver}\,\omega , \omega \in O \\ \bar{N} = \{1, \ldots , n\} &{} \text {set of certain copies of patients} \\ \tilde{N} = \{n+1, \ldots , 2n\}&{} \text {set of uncertain copies of patients} \\ \bar{r}_{jk} &{} \text {number of certain visits required by}\,j\hbox { in } W\hbox { for skill }k, j \in \bar{N}, k \in K\\ \tilde{r}_{jk} &{} \text {number of uncertain visits required by}\,j \hbox { in }W\hbox { for skill }k, j \in \tilde{N}, k \in K\\ a_{j} &{}\text {service (or assistance) time at patient}\,j, j \in \bar{N} \cup \tilde{N}\\ t_{ij} &{}\text {traveling time from node}\,i\hbox { to node }j, (i,j)\in A\\ H &{}\text {maximum number of caregivers who can visit each patient in}\, W \\ P_{j} &{}\text {set of patterns for}\, j, j \in \bar{N} \cup \tilde{N} \\ P &{}\text {overall set of patterns}. \end{array} \end{aligned}$$

Moreover, it makes use of the following families of variables:

$$\begin{aligned} z_{jp}= & {} \left\{ \begin{array}{ll} 1 \text{ if } \text{ pattern } p \text{ is } \text{ assigned } \text{ to } j \\ 0 \text{ otherwise } \end{array} \right. \quad j \in \bar{N} \cup \tilde{N}, p \in P_j\\ u_{\omega j}= & {} \left\{ \begin{array}{ll} 1 \text{ if } \text{ caregiver } \omega \text{ is } \text{ assigned } \text{ to } j \\ 0 \text{ otherwise } \end{array} \right. \quad \omega \in O, j\in \bar{N}\\ x_{ij}^{\omega d}= & {} \left\{ \begin{array}{ll} 1 \text{ if } \text{ caregiver } \omega \text{ travels along } (i,j) \text{ on } \text{ day } d \\ 0 \text{ otherwise } \end{array} \right. \quad (i,j) \in A, d \in W, \omega \in O_d\\y_{ij}^d \ge 0 &\quad \hbox {the number of nodes visited after }i\hbox { by a caregiver moving along }(i,j)\hbox { on day }d, d \in W\\ Cons_{jt}= & {} \left\{ \begin{array}{ll} 1 \text{ if } \text{ all } \text{ visits } \text{ to } \text{ patient } j \text{ are } \text{ scheduled } \text{ in } \text{ time } \text{ slot } t\\ 0 \text{ otherwise. } \end{array} \right. \quad j\in \bar{N}, t\in T \end{aligned}$$

Using the notation and the families of variables introduced above, the feasible set of the Consistent Robust Home Care Problem can be modeled as follows. Notice that the continuity of care constraints and the arrival time constraints are referred to by the same numbering used in Sect. 4.

21 $$\begin{aligned}&\sum _{(i,j) \in A}\sum _{\omega \in O_d}x_{ij}^{\omega d} \le \sum _{\begin{array}{c} p \in P_j:\\ p(d) \ge 1 \end{array}}z_{jp} \quad \forall j \in \bar{N} \cup \tilde{N}, \forall d \in W \end{aligned}$$

22 $$\begin{aligned}&\sum _{(i,j) \in A}\sum _{{\omega \in O_d: s_{\omega } \ge k}}x_{ij}^{\omega d} \ge \sum _{p \in P_j:p(d)=k}z_{jp} \quad \forall j \in \bar{N} \cup \tilde{N}, \forall d \in W, \forall k \in K\end{aligned}$$

23 $$\begin{aligned}&\sum _{p \in P_j}z_{jp} = 1 \quad \forall j \in \bar{N} \cup \tilde{N}\end{aligned}$$

24 $$\begin{aligned}&u_{\omega j} \le \sum _{(i,j) \in A} \sum _{d \in W} x_{ij}^{\omega d}+\sum _{(i,j+n) \in A} \sum _{d \in W} x_{i,j+n}^{\omega d}\quad \forall j \in \bar{N}, \forall \omega \in O\end{aligned}$$

25 $$\begin{aligned}&\sum _{(i,j) \in A}x_{ij}^{\omega d} = \sum _{(j,i) \in A}x_{ji}^{\omega d} \quad \forall j \in \bar{N} \cup \tilde{N}, \forall d \in W, \forall \omega \in O_d \end{aligned}$$

26 $$\begin{aligned}&\sum _{(0,j) \in A} y_{0j}^d = \sum _{j\in N} \sum _{p \in P_j:p(d)\ge 1} z_{jp} \quad \forall d \in W \end{aligned}$$

27 $$\begin{aligned}&\sum _{(i,j) \in A} y_{ij}^d - \sum _{(j,i) \in A} y_{ji}^d = \sum _{p \in P_j:p(d)\ge 1} z_{jp} \quad \forall j \in \bar{N} \cup \tilde{N}, d \in W\end{aligned}$$

28 $$\begin{aligned}&y_{ij}^d \le 2n\sum _{\omega \in O_d}x_{ij}^{\omega d} \quad \forall (i,j) \in A, d \in W \end{aligned}$$

1 $$\sum\limits_{{\omega \in O_{d} }} {\left( {\sum\limits_{{(i,j) \in A}} {x_{{ij}}^{{\omega d}} } + \sum\limits_{{(i,j + n) \in A}} {x_{{i(j + n)}}^{{\omega d}} } } \right)} \le 1\quad \forall j \in \bar{N},\forall d \in W$$

2 $$Cons_{{jt}} \le 1 - \left( {\sum\limits_{{\omega _{{\tilde{t}}} \in O,\tilde{t} \ne t}} {u_{{\omega _{{\tilde{t}}} j}} } /H} \right)\quad \forall j \in \bar{N},t \in T$$

3 $$\begin{aligned}&Cons_{jt} \ge \sum _{\omega _t \in O} u_{\omega _t j}/H\quad \forall j \in \bar{N}, t \in T \end{aligned}$$

4 $$\begin{aligned}&x_{ij}^{\omega d} \le u_{\omega j} \quad \forall j \in \bar{N}, \forall (i,j) \in A, \forall d \in W, \forall \omega \in O_d \end{aligned}$$

5 $$\begin{aligned}&x_{i,j+n}^{\omega d} \le u_{\omega j} \quad \forall j \in \bar{N}, \forall (i,j+n) \in A, \forall d \in W, \forall \omega \in O_d \end{aligned}$$

6 $$\begin{aligned}&\sum _{\omega \in O} u_{\omega j} \le H \quad \forall j \in \bar{N} \end{aligned}$$

7 $$\begin{aligned}&R^{\omega d}_{S\Gamma } \le D_{\omega } \quad \forall d \in W, \forall \omega \in O_d \end{aligned}$$

Constraints (21) state that at most one caregiver per day can visit *j*, if a visit has been scheduled on that day for node *j*. Constraints (22) guarantee that, on day *d*, for each skill *k* exactly one caregiver, of adequate skill, must visit *j* if a service of that skill has been scheduled for *j* on day *d*. In particular, if two skill levels are considered, caregivers of skill 1 can perform only visits of skill 1, whereas caregivers of skill 2 can perform both types of visits. Constraints (23) ensure that exactly one pattern is assigned to each patient. Notice that a pattern is assigned to each patient and also to its uncertain copy, in order to schedule all the certain and all the uncertain requests of each patient. The design variables $x_{ij}^{\omega d}$ thus model a set of tours including all the certain and all the uncertain requests. Constraints (24) fix the caregiver-patient assignment variable $u_{\omega j}$ to zero if caregiver $\omega$ does not visit neither the certain copy of patient *j* nor its uncertain copy ($j+n$) in the planning horizon. Constraints (25) are the classical flow conservation constraints on the routing variables. Constraints (26) and (27) are the flow conservation constraints on the auxiliary *y* variables, which are introduced to avoid subtours. They also guarantee the correct linking between scheduling and flow variables. Constraints (28) link together routing and flow variables. Constraints (1) impose that, on each day and for each patient *j*, at most one caregiver can visit patient *j* (either node *j* or node $j+n$). Constraints (2)-(3) model the consistency requirements. That is, they guarantee that all the caregivers assigned to a patient are related to the same daily time slot. Together with (2)-(3), constraints (4)-(6) express the care continuity requirement: (4) and (5) guarantee that a caregiver can visit a patient (precisely, the certain or the uncertain copy of a patient) only if assigned to that patient (linking between routing and caregiver assignment variables), while (6) bound the number of caregivers that can visit each patient during the time horizon. As an enhancement, constraints (24) force $u_{\omega j}$ to zero if caregiver $\omega$ never visits patient *j* during the week.

Finally, constraints (7) guarantee that the length of each sequence-preserving $\Gamma$-tour does not exceed the workday length of the assigned caregiver. More precisely, for each day *d* and each caregiver $\omega \in O_d$, $R^{\omega d}_{S\Gamma }$ represents the maximum length amongst all the sequence-preserving $\Gamma$-tours generated by the tour of $\omega$ on day *d*, say $\tau$. By imposing that such a maximum length cannot exceed the workday duration of $\omega$, we ensure that all the sequence-preserving $\Gamma$-tours generated by $\tau$ satisfy the length of $\omega$.

As in Cappanera et al. (2018), the length of each sequence-preserving $\Gamma$-tour is upper approximated by means of the following inner problem which makes use of the decision variables $v_{j}^{\omega d}$, $\forall j \in \tilde{N}$, $d \in W$, $\omega \in O_d$. Variable $v_{j}^{\omega d}$ takes on value 1 if the uncertain node $j \in \tilde{N}$ is visited on day *d* by caregiver $\omega$ (i.e. *j* belongs to a sequence-preserving $\Gamma$-tour of $\omega$ on day *d*), 0 otherwise. Precisely, for each day *d* and each caregiver $\omega \in O_d$, $R^{\omega d}_{S\Gamma }$ is approximated by $\bar{R}^{\omega d}_{S\Gamma }$, which is defined by the following model:

29 $$\begin{aligned} \bar{R}_{{S\Gamma }}^{{\omega d}} & = \sum\limits_{{(i,j) \in A:i,j \in \bar{N}^{ + } }} {(t_{{ij}} + a_{j} )} \cdot x_{{ij}}^{{\omega d}} + \sum\limits_{{(i,j) \in A:i \in \tilde{N},j \in \bar{N}^{ + } }} {(t_{j}^{{\max }} + a_{j} )} \cdot x_{{ij}}^{{\omega d}} \\ & \quad + \max \left\{ \begin{gathered} \sum\limits_{{(i,j) \in A:i \in \bar{N}^{ + } ,j \in \tilde{N}}} {(t_{{ij}} + a_{j} )} \cdot x_{{ij}}^{{\omega d}} \cdot v_{j}^{{\omega d}} \hfill \\ + \sum\limits_{{(j,i) \in A:i \in \bar{N}^{ + } ,j \in \tilde{N}}} {(t_{{ji}} - t_{i}^{{\max }} )} \cdot x_{{ji}}^{{\omega d}} \cdot v_{j}^{{\omega d}} + \sum\limits_{{(i,j) \in A:i \in \tilde{N},j \in \tilde{N}}} {(t_{j}^{{\max }} + a_{j} )} \cdot x_{{ij}}^{{\omega d}} \cdot v_{j}^{{\omega d}} \hfill \\ \end{gathered} \right\} \\ \end{aligned}$$

30 $$\begin{aligned}&\sum _{j \in \tilde{N}} v_{j}^{\omega d} \le \Gamma \end{aligned}$$

31 $$\begin{aligned}&v_{j}^{\omega d} \in \{0, 1\} \quad \forall j \in \tilde{N}. \end{aligned}$$

where $\bar{N}^{+}$ denotes the set of certain patients plus the depot node. Given a complete tour $\tau$, modelled by the $x_{ij}^{\omega d}$, and a sequence-preserving $\Gamma$-tour $\pi$ generated from it, whose uncertain selected nodes are defined by (30)-(31), the objective function in (29) estimates the length of $\pi$ by considering exactly the contribution due to the arcs linking certain nodes as well as the depot (first summation in (29)), and also the contribution of the arcs linking certain nodes and depot to selected uncertain copy nodes, i.e. to nodes $j \in \tilde{N}$ such that $v_{j}^{\omega d}=1$ (third and fourth summation in (29)). In addition, for the arcs (*i*, *j*) not in the tour and for those connecting two selected uncertain nodes, the traveling time of (*i*, *j*) is estimated from above via $t_{j}^{max}$, where $t_{j}^{max} = \max _{(i,j) \in A}\{t_{ij}\}$ is the maximum travel time amongst all arcs entering *j*, for each node *j*. Therefore, problem (29)-(31) looks for a maximum length sequence-preserving $\Gamma$-tour based on the just stated over-estimation of the tour length. The correctness of the upper approximation derives from Proposition 1 in Cappanera et al. (2018). Then, the inner problem can be dualized and inserted in the complete model via standard techniques used in Robust Optimization (Bertsimas and Sim (2004)).

The presented formulation to the Consistent Robust Home Care Problem has been experimented by using a social equity criterion, aiming at balancing the caregiver workloads. This kind of criterion was also investigated in Cappanera and Scutellà (2015), Yalçındağ et al. (2016) and Cappanera et al. (2018). Precisely, we minimize the maximum caregiver utilization factor, where the *caregiver utilization factor* is expressed as the total workload of the caregiver during the planning horizon in the worst scenario, over his maximum possible workload.

## References

[CR2] Borsani V, Matta A, Beschi G, Sommaruga F (2006) A Home Care scheduling model for human resources. In: 2006 international conference on service systems and service management, Troyes, pp 449-454. 10.1109/ICSSSM.2006.320504

[CR3] Bennett AR, Erera AL (2011). Dynamic periodic fixed appointment scheduling for home health. IIE Trans Healthcare Syst Eng.

[CR4] Bertsimas D, Sim M (2004). The price of robustness. Operat Res.

[CR5] Bryant PA, Rogers BA, Cowan R, Bowen AC, Pollard J (2020). Planning and clinical role of acute medical Home Care services for COVID-19: consensus position statement by the Hospital-in-the-Home Society Australasia. Int Med J.

[CR6] Cappanera P, Scutellà MG (2015). Joint assignment, scheduling, and routing models to Home Care optimization: A pattern-based approach. Transp Sci.

[CR7] Cappanera P, Scutellà MG (2017) Pattern generation policies to cope with robustness in Home Care. Springer Proceedings in Mathematics & Statistics. In: Proceedings of the International Conference on Health Care. Systems Engineering, vol 210, pp 257–268

[CR8] Cappanera P, Scutellà MG, Nervi F, Galli L (2018). Demand uncertainty in Robust Home Care optimization. Omega Int J Manage Sci.

[CR9] Cappanera P, Requejo C, Scutellà MG (2020). Temporal constraints and device management for the Skill VRP: mathematical model and lower bounding techniques. Comput Oper Res.

[CR10] Carello G, Lanzarone E (2014). A cardinality-constrained robust model for the assignment problem in Home Care services. Europ J Oper Res.

[CR11] Carello G, Lanzarone E, Laricini D, Servilio M (2017). Handling time-related demands in the Home Care nurse-to-patient assignment problem with the implementor-adversarial approach. Springer Proc Math Stat.

[CR12] Chen X, Thomas BW, Hewitt M (2012). Multi-period technician scheduling with experience-based service times and stochastic customers. Comput Oper Res.

[CR13] Cissé M, Yalçındağ S, Kergosien Y, Şahin E, Lenté C, Matta A (2017). OR problems related to home health care: a review of relevant routing and scheduling problems. Oper Res Health Care.

[CR14] Coloma E, Nicols D (2020). Hospital at Home units in the post-COVID 19 era. Europ J Clin Invest.

[CR1] Di Mascolo M, Espinouse M-L, El Zied H (2017). Planning in home health care structures: a literature review. IFAC-PapersOnLine.

[CR15] Demirbilek M, Branke J, Strauss A (2018). Dynamically accepting and scheduling patients for home healthcare. Health Care Manag Sci.

[CR16] Demirbilek M, Branke J, Strauss A (2019). Home healthcare routing and scheduling of multiple nurses in a dynamic environment. Flexible Serv Manuf J.

[CR17] Du G, Zheng L, Ouyang X (2019). Real-time scheduling optimization considering the unexpected events in home health care. J Combin Optim.

[CR18] Duque PAM, Castro M, Sörensen K, Goos P (2015). Home Care service planning. The case of Landelijke Thuiszorg. Europ J Oper Res.

[CR19] Eveborn P, Flisberg P, Rönnqvist M (2006). Laps care - and operational system for staff planning of Home Care. Europ J Oper Res.

[CR20] Feillet D, Garaix T, Lehuédé F, Péton O, Quadri D (2014). A new consistent vehicle routing problem for the transportation of people with disabilities. Networks.

[CR21] Fikar C, Hirsch P (2017). Home health care routing and scheduling: a review. Comput Oper Res.

[CR22] Gamst M, Jensen TS, Klatte D, Lthi H-J, Schmedders K (2012). A branch-and-price algorithm for the long-term Home Care scheduling problem. Operations Research Proceedings.

[CR23] Groër C, Golden B, Wasil E (2009). The consistent vehicle routing problem. Manuf Serv Oper Manage.

[CR24] Hewitt M, Nowak M, Nataraj N (2016). Planning strategies for home health care delivery. Asia-Pacific J Oper Res.

[CR25] Holte M, Mannino C (2013). The implementor/adversary algorithm for the cyclic and robust scheduling problem in health-care. Europ J Oper Res.

[CR26] Kovacs AA, Golden BL, Hartl RF, Parragh SN (2014). Vehicle routing problems in which consistency considerations are important: a survey. Networks.

[CR27] Kovacs AA, Parragh SN, Hartl RF (2014). A template-based adaptive large neighborhood search for the consistent vehicle routing problem. Networks.

[CR28] Kovacs AA, Golden BL, Hartl RF, Parragh SN (2015). The generalized consistent vehicle routing problem. Transp Sci.

[CR29] Kovacs AA, Parragh SN, Hartl RF (2015). The multi-objective generalized consistent vehicle routing problem. Europ J Oper Res.

[CR30] Lin C-C, Hung L-P, Liu W-Y, Tsai M-C (2018). Jointly rostering, routing, and rerostering for home health care services: a harmony search approach with genetic, saturation, inheritance, and immigrant schemes. Comput Ind Eng.

[CR31] Liu R, Xie X, Garaix T (2014). Hybridization of tabu search with feasible and infeasible local searches for periodic home health care logistics. Omega.

[CR32] Liu R, Yuan B, Jiang Z (2018). A branch-and-price algorithm for the home-caregiver scheduling and routing problem with stochastic travel and service times. Flexible Serv Manuf.

[CR33] Nguyen TVL, Toklu NE, Montemanni R (2015). Matheuristic optimization for robust home health care services. Lecture Notes Manage Sci.

[CR34] Nickel S, Schröder M, Steeg J (2012). Mid-term and short-term planning support for home health care services. Europ J Oper Res.

[CR35] Rasmussen MS, Justesen T, Dohn A, Larsen J (2012). The Home Care crew scheduling problem: preference-based visit clustering and temporal dependencies. Europ J Oper Res.

[CR36] Redjem R, Marcon E (2016). Operations management in the Home Care services: a heuristic for the caregivers’ routing problem. Flexible Serv Manuf J.

[CR37] Rodriguez C, Garaix T, Xie X, Augusto V (2015). Staff dimensioning in homecare services with uncertain demands. Int J Prod.

[CR38] Sungur I, Ren Y, Ordonez F, Dessouky M, Zhong H (2010). A model and algorithm for the courier delivery problem with uncertainty. Transp Sci.

[CR39] Tarantilis C, Stavropoulou F, Repoussis P (2012). A template-based Tabu search algorithm for the consistent vehicle routing problem. Exp Syst Appl.

[CR40] Trautsamwieser A, Hirsch P (2014). A branch-price-and-cut approach for solving the medium-term home health care planning problem. Networks.

[CR41] Souyris S, Cortés CE, Ordóñez F, Weintraub A (2013). A robust optimization approach to dispatching technicians under stochastic service times. Optim Lett.

[CR42] Yalçındağ S, Cappanera P, Scutellà MG, Matta A, Şahin E (2016). Pattern-based decompositions for human resource planning in home health care services. Comput Oper Res.

[CR43] Yuan B, Liu R, Jiang Z (2014) Home health care crew scheduling and routing problem with stochastic service times. In: Proceedings of 2014 IEEE international conference on automation science and engineering, pp 564–560

